# Systematic review of the psychometric properties of quality-of-life outcome measures used with adult inpatient psychiatric populations

**DOI:** 10.1007/s11136-026-04241-9

**Published:** 2026-04-01

**Authors:** Sinead McLernon, Scott Steen, Claire Bone, Jaime Delgadillo

**Affiliations:** 1 University of Sheffield, Sheffield, UK; 2 Kings College London, London, UK

**Keywords:** COSMIN, Quality of life, Measurement properties, Psychiatric inpatient, Reliable change index

## Abstract

**Purpose:**

To systematically review available quality of life (QoL) measures used in adult psychiatric inpatient settings following the Consensus-based Standards for the selection of health Measurement Instruments (COSMIN) method.

**Methods:**

Systematic searches across four databases were conducted following a pre-registered review protocol: MEDLINE, PsycINFO, Scopus, and CINAHL. Forward and backwards citation searches were also conducted. Psychometric properties (content validity, structural validity, internal consistency, cross-cultural validity, test-retest reliability, measurement error, criterion validity, construct validity, and responsiveness) were assessed against the COSMIN criteria. A narrative synthesis was employed to integrate the results. The GRADE approach was used to assess overall certainty of the evidence. Reliable change indices (RCI) were calculated for QoL measures where sufficient data were available.

**Results:**

Twenty-two QoL measures were included in the review, from 38 studies. Most measures had evidence of good structural validity and internal consistency in this clinical population. However, no outcome measure covered all measurement properties defined by COSMIN and a limited number covered content validity.

**Conclusion:**

Overall, a small subset of QoL measures demonstrated adequate performance across multiple psychometric properties, including: S-QOL-41, ReQoL, SF-36, and MHQOL.

**Supplementary Information:**

The online version contains supplementary material available at 10.1007/s11136-026-04241-9.

## Abstract (plain language summary)

1. Why is this study needed?

Patient-reported outcome measures allow clinicians and patients to monitor change over time and to identify areas that the patient might want to focus on. QoL measures can provide a person-centred view of recovery including domains other than symptom reduction.

2. What is the key problem/issue/question this manuscript addresses?

To date, little is known about the psychometric validity and reliability of QoL measures applied in psychiatric inpatient care settings.

3. What is the main point of your study?

To our knowledge, there are currently no reviews that summarise the psychometric properties of all available QoL outcome measures in inpatient psychiatric settings. This review provides a comprehensive overview of available measures, highlighting their strengths and limitations.

4. What are your main results, and what do they mean?

There is currently no QoL outcome measure which covers all psychometric properties as defined by COSMIN guidelines for the inpatient psychiatric population.However, several of the reviewed outcome measures showed promising evidence.

**Supplementary Information:**

The online version contains supplementary material available at 10.1007/s11136-026-04241-9.

## Introduction

In mental health care, patient-reported outcome measures (PROMs) are commonly used to monitor changes over time. When used effectively, they can inform care planning [70] and enhance patient outcomes [13]. Beyond symptom reduction, PROMs are particularly valuable for monitoring changes in Quality of Life (QoL) [74]. QoL refers to the standard of health, well-being, comfort, and happiness experienced by an individual or group [69]. The World Health Organisation (WHO) describes Quality of Life (QoL) as an individual’s perception of their position in life within context, culture and values [77]. Improvements in QoL can, therefore, be significant for individuals, and enhancing QoL is often a primary goal in mental health care.

Despite the significance of QoL measures in research and practice, researchers have not reached a universal consensus on its measurement (Blome & Augustine, [8]; [10, 16]). It is considered a complex, multidimensional construct often encompassing both objective and subjective life domains [21, 69]. While aggregate QoL scores provide a useful metric of global well-being, they can obscure meaningful variations within specific life areas and socio-demographics, potentially impacting accurate evaluations of health inequities. By focusing on individual domains including physical health (e.g., sleep and symptoms), psychological well-being (e.g., mood, self-esteem, and cognition), social integration (e.g., family relationships and appreciation by others), and functional independence (e.g., work, household duties, and financial situation), we might provide a more sensitive lens for population health assessment.

It is generally agreed that the content of QoL measures should be co-designed with patients to enhance content validity to the target population [5]. Therefore, measures used in community settings may not be suitable for addressing the problems faced by those in inpatient settings, where issues of autonomy, safety, and restrictive environment play a role. Psychiatric inpatient settings are defined as 24-hour services delivered in hospital settings for those whose mental health condition is severe, acute, or complex enough that community-based services are insufficient (NHS England, [51]). Research conducted in inpatient psychiatric settings can involve multiple settings and populations due to their dynamic and transitional nature [66]. Accordingly, given their dynamic quality, partial inpatient populations are also of interest in this review.

To evaluate whether an outcome measure is reliable and valid, its psychometric properties should be systematically assessed and reported [25]. Previous reviews have evaluated the psychometric properties of specific QoL measures, such as the EuroQoL-5D and Short Form-36, in patients with schizophrenia [56]. Additionally, Zuniga Le-Bert et al., [82] examined the Schizophrenia QoL Scale (SQLS) and revision four (SQLS-R4) alongside their psychometric properties. Van Krugten et al., [74] conducted a review which identified QoL outcome measures used in mental health settings. However, the psychometric properties of identified outcome measures weren’t evaluated. To our knowledge, there is no agreement on how quality of life should be measured or which instrument is best to use in a psychiatric inpatient setting [57].

This review aims to identify the QoL outcome measurement instruments (OMIs) used in adult psychiatric inpatient settings and to evaluate the adequacy of their psychometric properties. In this review, *psychometric properties* refer to content validity, structural validity, internal consistency, cross-cultural validity, test-retest reliability, measurement error, criterion validity, construct validity, and responsiveness, as defined in the COSMIN manual.

## Methods

The Preferred Reporting Items for Systematic Reviews and Meta-Analyses (PRISMA) updated COSMIN-OMI reporting guidelines [17] guided the review. The protocol was pre-registered with PROSPERO (protocol ID: CRD420250651806).

### Search strategy

Eligibility was assessed by using the PICOS framework (population, intervention, comparator, outcome and study design) [1]. The inclusion and exclusion criteria, which guided the development of the search strategy, are described in Table 1. The search was limited to papers published in the English language and grey literature was included. No time limits were applied to the search strategy. The search was conducted on April 18, 2025, across four databases: MEDLINE (Ovid), PsycINFO (Ovid), Scopus, and CINAHL. Searches on databases such as PsycINFO and Medline included medical subheadings (MeSH) terms and the option to “explode” specific terms, such as “quality of life” and “psychometrics”. A comprehensive search strategy is outlined in Appendix A. Forward and backwards citation searching was conducted based on eligible studies identified in preliminary steps of the selection process. Finally, a review of the reference list of the study by Van Krugten et al., [74] was conducted to build on this previous review, which identified QoL outcome measures used in mental health settings. The primary author selected papers according to the inclusion criteria by initially screening titles and abstracts, followed by a full-text review.

**Table 1:** Summary of inclusion and exclusion criteria

	Inclusion criteria		Exclusion criteria
Population	Adult psychiatric inpatients. This refers to individuals admitted to general adult psychiatric inpatient as opposed to specialist settings. For example, studies conducted in psychiatric inpatient wards that may include a mix of diagnoses (e.g., patients with eating disorders alongside other psychiatric conditions) are included. Studies should report on psychiatric inpatients. Studies that include mixed populations (for example, both inpatients and outpatients) are included, these studies may contribute valuable comparisons between groups (for example, discriminant validity). Studies exclusively involving outpatients are excluded. As the review is exploratory to identify knowledge gaps and aims to give a broad overview of available OMIs in this population, mixed populations are included. Results were critically assessed to reflect this. Different length of stay inpatients (acute, rehabilitation, and long stay patients) and different ward intensity are included (rehabilitation, acute, PICU).		Specialist inpatient settings are excluded such as specialist eating disorder units, specialist forensic units such as secure wards, learning disability wards and drug and detox wards. These settings focus on treating specific diagnostic or legal populations outside the scope of the review which focuses on general psychiatric inpatient with a mix of diagnosis. Studies that define the population as older adult or adolescent are excluded as the review is limited to general adult psychiatric inpatients. No specific age range was specified as countries define these categories differently. Instead, articles which defined their population as “older adult” or “adolescent” are excluded. Inpatients settings focused only on physical health, such as stroke rehabilitation units or general medical wards, are excluded, as they do not primarily provide psychiatric care.
Intervention	Papers which evaluate measurement properties of quality-of-life measurement tools, for example, validation studies.		Studies which do not report on or include measurement properties of quality-of-life outcome tool. Interventional studies, for example, those that use an outcome measure for pre- and post-intervention evaluation but do not explicitly evaluate measurement properties of the outcome measure.
Comparator	Using different measurement properties, such as reliability indices, the identified outcome measures are compared using the COSMIN OMI guideline for systematic reviews [17].		
Outcomes	The psychometric properties of QoL measures including content validity, structural validity, internal consistency, cross-cultural validity, test-retest reliability, measurement error, criterion validity, construct validity and responsiveness.		Outcome measurement tools that do not assess QoL are not included. For example, studies that report symptom severity outcome measures.
Study design	Studies that evaluate measurement properties of QoL outcome measures are included. This might include longitudinal design, cross-sectional design, and repeated measures. Content validity studies typically rely on qualitative evidence, as such, relevant qualitative studies are included.		Studies which do not evaluate or report the measurement properties of QoL outcome measures.

### Analysis

The COSMIN reporting guidelines [48] recommend evaluating outcome measurement instruments (OMIs) using six stages. OMIs are defined by COSMIN as instruments used to monitor changes in health over time [48].

#### Stage 1: data extraction

Relevant information was extracted and collated using COSMIN review management file. Data extracted included study characteristics (study setting, participants, diagnosis, design and methods, and outcome measures used), sample characteristics (age, gender, ethnicity, presenting problems, country), outcome measurement tool characteristics (domains, number of items, time to complete, common usage, related and paired measures, availability, licensing, and scoring procedure), and psychometric properties. Information including mean scores, standard deviations and Cronbach’s Alpha, was extracted to calculate the Reliable Change Index (RCI) [31] for each QoL measure.

#### Stage 2: evaluation

Each psychometric property identified in each study was scored against the COSMIN risk of bias assessment [48]. COSMIN risk of bias ratings are rated on a scale of Inadequate (I), Doubtful (D), Adequate (A) and Very Good (VG). A “very good” rating indicates the standard has been met using the preferred methods. An “inadequate” score indicates the standard has not been met, as the methods used are inadequate. Following COSMIN guidelines, the lowest score across all items for a psychometric property determines the overall rating.

#### Stage 3: rating study results

Each psychometric property was evaluated against the COSMIN criteria for good measurement properties, which were used to determine whether the study results were sufficient or not [48]. A score of (-) indicates that the psychometric property is insufficient; there is evidence that the psychometric property is of poor quality. A score of (?) indicates that an indeterminant rating has been made, which means that something has been done, but there is not enough information to rate the result. A score of (+) indicates the psychometric property is sufficient, and there is evidence that it is of good quality.

#### Stage 4: summarising the results

Multiple studies using the same QoL measure are summarised. At this stage, differences in results between studies were noted. For example, if some studies report sufficient psychometric properties for the OMI and others report insufficient ones, a narrative explanation was provided about the differences.

#### Stage 5: rating summarised results

Psychometric properties were given a final summarised rating of sufficient (+), insufficient (-), inconsistent (+/-) or indetermined (?). Content validity was evaluated based on relevance, comprehensibility, and comprehensiveness. This provided an overall conclusion for each OMI reported.

#### Stage 6: grade assessment

The Grading of Recommendations Assessment, Development, and Evaluation (GRADE) approach [27] was used to assess the certainty of the evidence for each psychometric property across the included studies, following the COSMIN guidelines [48]. This modified GRADE approach evaluates the body of evidence for factors that may warrant downgrading. Specifically, it considers the methodological quality of the studies, consistency, precision (sample size), and directness of the results (i.e., the population of interest). A final certainty rating of “high”, “moderate”, “low”, or “very low” was provided. Any final recommendations about the reviewed measures were based on the GRADE approach. COSMIN guidelines recommend assessing all nine psychometric properties before making a recommendation.

Finally, the reliable change index (RCI) [31] was calculated and reported using means, standard deviations, and Cronbach’s Alpha scores entered into a Shiny app developed to compute RCI (Evans, 1998).

## Results

**Fig. 1:**
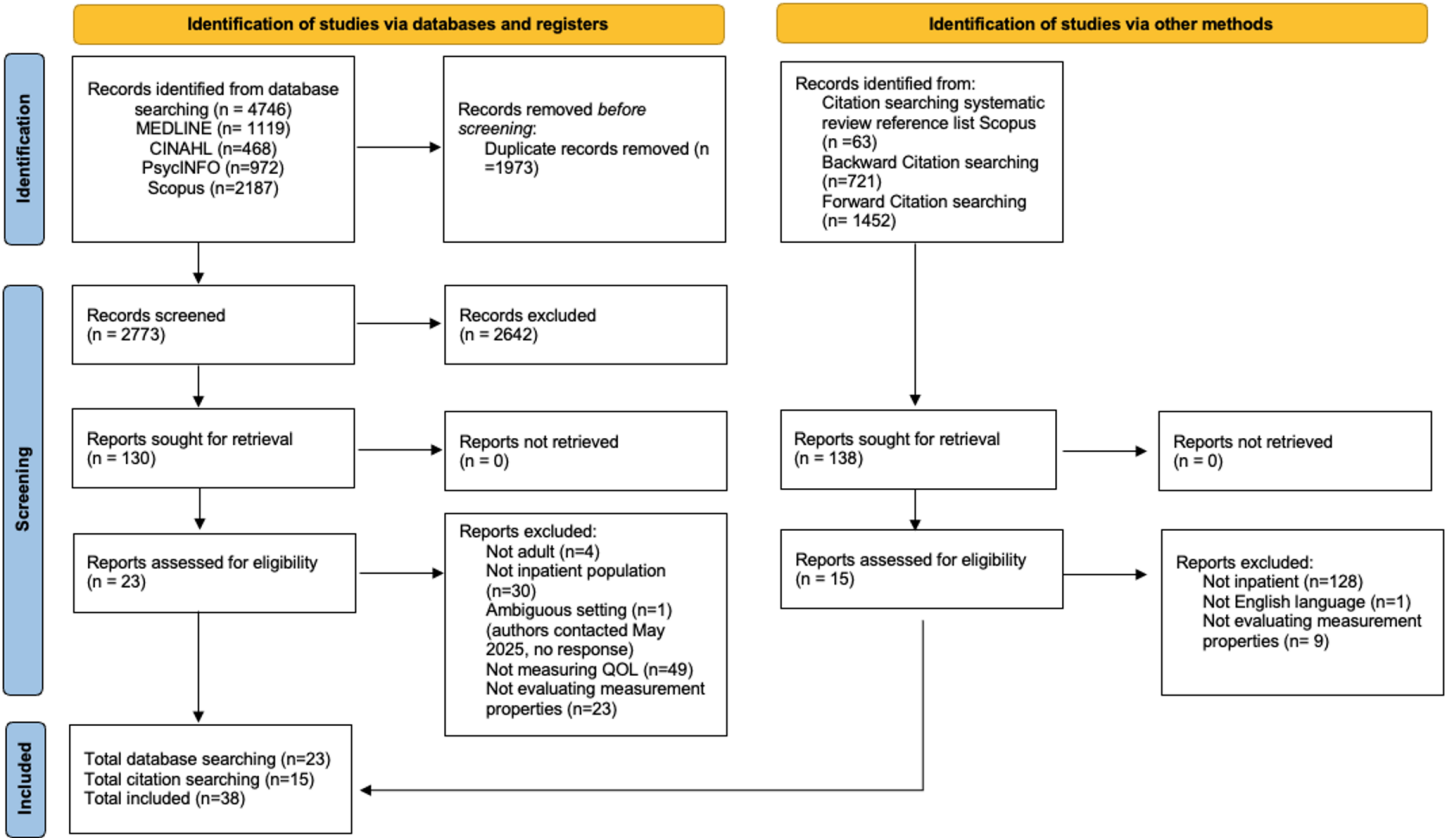
COSMIN-PRISMA flow diagram of study selection

Study selection is outlined in the COSMIN-PRISMA diagram (Fig. 1). A total of 2773 records were identified after duplicates were removed from the database searches. After title and abstract screening, 130 reports were sought for retrieval. After full-text screening, 23 reports were included in the review. One record by Mezzich et al., [46] was excluded due to the ambiguity of the inpatient setting. A further 2173 records were identified through forward and backwards citation searching, and 138 records were retrieved after title and abstract screening. A further 63 references were identified from a previous review of QoL instruments in mental health [74]. After full-text screening, 15 articles from citation searching were included. A random selection of full-text articles was independently screened by a second reviewer (10%) with 100% agreement. A total of 38 reports were included in the review, which included 22 outcome measures. Reasons for excluding studies are included in the COSMIN-PRISMA diagram.

### Study characteristics

Information on study characteristics was extracted (Table 2). OMI characteristics are available in the supplementary materials. Of the studies reviewed, the search did not return any studies that administered outcome measures by proxy. More information on the administration method is available in the supplementary materials. Outcome measures were organised together rather than listed alphabetically, as recommended by COSMIN. Studies reporting more than one QoL measure are included multiple times for each measure.

The mean age of participants ranged from 31.3 to 53.4 years, with most studies reporting average ages of participants in their 30’s. The proportion of male participants varied across studies, ranging from 29 to 79%, with an average of 63.4%. The duration of illness was reported, ranging from 8.5 to 26.5 years, with an average of 15.2 years. Most studies were conducted in Europe and Asia and were published in English. All studies included at least a partial inpatient population. Some studies reported a mixed sample and included additional settings such as board and care homes, community homes, community care, residential care, halfway houses, primary and secondary care and comparison groups of professionals and the public. Studies which included a mixed sample received a lower score for the quality of evidence (QOE) for “indirectness”.

Sample sizes ranged from *n* = 21 to *n* = 4266, with variations within studies for each measurement property assessed. A range of study designs were employed, including cross-sectional, longitudinal, prospective, group comparison, repeated measures, multi-trait scaling analysis, and between-subjects designs. Information on QoL instrument characteristics is in Supplementary Table 1.

**Table 2:** Study characteristics

OMI	Outcome version	Reference	Country (Language, if reported)	Setting	Design	Demographic characteristic patients			Illness characteristics	
						Mean (SD) age	% male	Ethnicity	Diagnosis %	Duration (years)
LQoLI-BREF	Lehman et al., [40]	Anderson et al., [2]	Canada (English)	Inpatient (93.6%) rehabilitation 3 participants were outpatient	NR suggestive of longitudinal/repeated measures	40.12 (8.6)	61	100% White	Schizophrenia 74 Bipolar 15 psychosis 11 major depression 2	3.91
2 LQoLI Full	Lehman, [38]	Russo et al., [63]	USA (English)	Acute inpatient	Longitudinal	35.6 (11.4)	61.1	White (77.3%) African-American (15.7%) Asian (2.9%) and Latino (2%)	Severely ill 100	2
2 LQoLI Full	Lehman, [38]	Lehman, [38]	USA (English)	Inpatient 21,1%, outpatient 19.6%, and board and care homes	NR suggestive of repeated measures	57.5	47	74.8–90.2% White	Inpatient population schizophrenia 76.8, Affective disorder 13.1 Alcoholism 6.1 drug abuse 1 Organic brain syndrome 4 intellectual disability 9.1 Personality disorder 5.1	23.7
2 LQoLI Full	Quality of Life Interview – core version Lehman, [39]	Goodwin & Madell, [26]	UK (English)	Inpatient 23%, Community home 67%, and Community own home 15%	NR, suggestive of Randomised repeated measures	51	52	NR	Schizophrenia and other mental health difficulties not defined 100	23
3 Q-LES-Q	Endicott et al., [18]	Bishop et al., [7]	USA (English)	Admitted to either a residential community treatment centre or a University psychiatric hospital	NR- suggestive of cross-sectional design	18–65^a^a^	45	Caucasian: 50% African American: 24% Hispanic: 7% Asian: 1% No Data: 19%	Schizophrenia 34.5 Bipolar Disorder 16.6 Psychosis NOS 21.6 Major Depression 19.4 Other 7.9	NR
3 Q-LES-Q	Endicott et al., [18]	Ritsner et al., [62] (10 items removed)	Isreal (Hebrew)	Inpatient 35.5% and outpatient 17.6% professionals 46.7%	Not reported, suggestive of a between subject’s design	38.9 (10.1)	79	NR	Schizophrenia 74 Schizoaffective disorder 17 Mood disorder 9	14.1
4 Q-LES-Q SF	Based on [18] (general activities subscale)	Pitkanen et al., [58]	Finland (Finish)	Acute inpatient	Not reported, suggestive of repeated measures	38 (13)	59	NR	Schizophrenia 41 unspecified non-organic psychotic disorder 29 schizoaffective disorder, 16 acute and transient psychotic disorder 6 persistent delusional disorder 5 schizotypal disorder 2 other non-organic psychotic disorder (F28) 1	NR
5 LQOLP	Oliver et al., [55]	Ritsner et al., [62] (only 27 items considered)	Isreal (Hebrew)	Inpatient 35.5% and outpatient 17.6% professionals 46.7%	NR, suggestive of a cross sectional comparative design	38.9 (10.1)	79	NR	Schizophrenia 74 Schizoaffective disorder 17 Mood disorder 9	14.1
5 LQOLP	Oliver et al., [55]	Kaiser et al., [32]	England and Germany (English and German)	In (55.2%) and outpatient (54.8%)	NR, suggestive of cross sectional comparative design	53.4 (14)	59	NR	neuroleptics (being treated for psychosis) also depression bipolar and schizophrenia (% NR)	12.32
5 LQOLP	Oliver et al., [55]: validation of LQOLP-EU version	Gaite et al., [24]	Netherlands, Denmark, Uk, Italy (Dutch, Danish, English, Spanish, Italian)	Inpatient, outpatient and community	Test-retest study (longitudinal)	18–65	NR	NR	Schizophrenia, diagnosis F20-F25 100	NR
5 LQOLP	Oliver et al., [55] Swedish translation	Hansson et al., [28]	Sweden (Swedish)	Inpatient	Cross-sectional (longitudinal)	31.3	51.7	NR	Psychosis 100	NR
6 S-QOL-18	S-Qol-18 validation study	Boyer et al., [9]	France (French)	In 28.6% and outpatient 71.3%	Repeated measures (longitudinal)	36.5 (10.8)	70	NR	Schizophrenia 100	13.8
7 SQOL-41	S-SQOL-41 development study, based on [4]	Auquier et al., [5]	France (French)	In and outpatient 50%	Randomised test retest (longitudinal)	37.3 (10.9)	68	NR	Schizophrenia paranoid 46.9 disorganized 14 undifferentiated 15.9 residual 21.7	13.9
7 SQOL-41	S-QOL-C Auguier et al., [5]	Chou et al., [12]	China (Chinese)	Inpatient	Prospective study	37.9	70.7	NR	Schizophrenia 100	NR
8 QLI	Frisch, (1993)	Angstman et al., [3]	USA (English)	Inpatient	NR	37.8 (11.6)	41	88% Caucasian and 18% Native American	schizophrenia 29 depression 23 bipolar disorder 20 substance disorders 14 personality disorders 11 other 3	NR
9 QLIS	Development study	Franz et al., [22]	Germany (German)	Inpatient 67%, outpatient 31%, general population	Cross sectional	37.3–40.7 (9,8-12.9)	41–50	NR	Schizophrenia 100	25.2–30.7 months
9 QLIS	Franz et al., 23	Franz et al., [23]	Germany (German)	Inpatient 39% and outpatient 61%	Cross sectional	37.4 (9.8)	54	NR	Schizophrenia 100	12
10 How Are You? Scale	Development study	Katsavdakis et al., [34]	UK (English)	Inpatient compared to professionals	Repeated measures within subjects and between groups	33.6 (11.4)	33	NR	alcohol dependency 16 anxiety disorder 14 depressive 24 bipolar disorder 9 psychotic disorder 12 eating disorder 12 all remaining diagnostic categories 13	16 days
11 QLS	Development study	Nair et al., [49]	Singapore (Not reported)	Inpatient	NR suggestive of Repeated measures	49.39 (10.58)	54	Chinese (765), Malay 8% Indian 12%, other, 4%	Schizophrenia 100	7.68
12 Sf-36	Ware, [75]	Newnham et al., [50] (only subscales related to mental health)	Australia (Not stated)	Private inpatient hospital	NR suggestive of Repeated measures	13–87	29	NR	affective/mood 66 anxiety 22 schizophrenia 4 substance 5	2
12 Sf-36	Ware, [75]	Pukrop et al., [60]	Germany (NR)	Inpatient	NR suggestive of Between subjects longitudinal design	33.7 -43.45(9.6-12.42)	38.9–51.6	NR	Schizophrenia 16 Depression 21 Healthy controls 63	6.7
12 Sf-36	Ware, [75]	Nishiyama et al., [52]	Japan (Japanese)	Inpatient	Multi trait scaling analysis	50 (14.7)	53.6	NR	Schizophrenia 77.4 Bipolar 4.4 Major depressive Disorder 2.9 Dementia 5.8 intellectual disability 7.3	4.8 hospital
12 Sf-36	Ware et al., [76]	Su et al., [67]	Taiwan (Chinese)	Inpatient	NR suggestive of Repeated measures and cross sectional	49.1 (7.85)	66	NR	Schizophrenia 100	26.5
12 Sf-36	Ware et al., [75]	Tunis et al., [71]	USA (English)	In and outpatient	NR suggestive of Repeated measures and cross sectional	39.29 (11.32)	70	75% White	Schizophrenia 80 Schizoaffective disorder 20	24
13 WHOQOL-BREF	WHOQOL-BREF Taiwan version Yao et al., [81] (2 additional items)	Su et al., [67]	Taiwan (Chinese)	Inpatient	NR suggestive of Repeated measures	49.1 (7.85)	66	NR	Schizophrenia 100	26.5
13 WHOQOL-BREF	WHO- QOL Group, [78]	Oliveira et al., [54]	Portugal (Portuguese)	Inpatient (34%) and outpatient (66%)	Cross-sectional	43.15 (12.38)	57	NR	Schizophrenia 36.5 Bipolar 8.2 Anxiety 3.7 Personality Disorders 14.1 Depressive Disorder 20.1 Psychotic Disorder 10.4	NR
13 WHOQOL-BREF	WHO- QOL Group, [78]	Norholm & Bech, [53]	Denmark (Danish)	Inpatient	NR suggestive of cross sectional	39(10.9)	58.9	NR	Schizophrenia 100	69 days hospital (+/- 64 days)
13 WHOQOL-BREF	WHOQOL-BREF-HK Leung et al., [41]	Chan et al., [11]	Hong Kong (not specified, Hong Kong version used)	Inpatient (31.3%), long stay care homes and halfway houses	Matched group comparison	49.84 (7.88)	59.7	NR	Schizophrenia 100	26
13 WHOQOL-BREF	WHOQOL Group, [78]	Willige et al., [72]	Netherlands (not described)	Inpatient and out patient	longitudinal	36 (11.2)	55	NR	Schizophrenia 100	11
14 MSQOL	Pukrop et al., [59]	Pukrop et al., [60]	Germany (NR)	Inpatient	Between subjects longitudinal design	33.7 -43.45(9.6-12.42)	38.9–51.6	NR	Schizophrenia 16 Depression 21 Healthy controls 63	6.7
15 EQ-5D	EuroQoL Group, [20] Descriptive scale only	Pikanen et al., [58]	Finland (Finish)	Acute inpatient	NR suggestive of cross sectional comparative design	38 (13)	59	NR	Schizophrenia 41 unspecified non-organic psychotic disorder 29 schizoaffective disorder 16 acute and transient psychotic disorder 6 persistent delusional disorder 5 schizotypal disorder 2 other non-organic psychotic disorder (F28) 1	NR
15 EQ-5D	EuroQoL Group, [19]	Willige et al., [72]	Netherlands (NR)	Inpatient and out patient	longitudinal	36 (11.2)	55	NR	Schizophrenia 100	11
16 MHQOL	Van Krugten et al.,[73] (validated in a Persian context) MHQOL Persian version (VAS and 7D)	Ebadi & Rezaiye, [15]	Iran (Farsi)	Inpatient	NR suggestive of Cross sectional and Repeated measures	18+	61	NR	Major depressive disorder 28.7 Bipolar 27.3 PTSD 28.7 Schizophrenia 15.3	NR
17 SF-12	Turkish form of SF-12 v2 Maruish et al., [44].	Soysal Gunduz et al., [65]	Turkey (Turkish)	Physical health ward and Psychiatric ward (28.4%)	Cross-sectional design	43.5 (14.4)	49		Physical and Mental health, bipolar disorder, Major depression, PTSD, OCD, Anxiety, Psychosis (% NR)	NR
18 ReQoL	Development study	Keetharuth et al., [35]	UK (English)	General practices (6–27%), IAPT (6%), Secondary care, Inpatient (3–13%) out patient (46–57%), and Community	NR suggestive of Cross sectional and longitudinal	16–65^a^b^	NR	White (85–86%), Black and minority ethnic (9–13%)	Common mental health disorders, Schizophrenia, other psychotic disorders, bipolar, personality disorders, others and missing data (% NR)	NR
19 SQLS	Wilkinson et al., [79] (Japanese version)	Kaneda et al., [33]	Japan (Japanese)	Inpatient	NR suggestive of Cross sectional,	46.6 (11.3)	45	NR	Schizophrenia 100	22.9
19 SQLS	Wilkinson et al., [79] (revision 4)	Kuo et al., [36]	Taiwan (Chinese)	Inpatient (50%) and outpatient (50%)	NR suggestive of Repeated measures	36.9 (8.82)	75	NR	Schizophrenia 100	12.5
19 SQLS	Wilkinson et al., [79] (revision 4)	Kuo et al., [37]	Taiwan (Chinese)	Inpatient (50%) and outpatient (50%)	NR suggestive of Cross sectional,	36.9 (8.82)	75	NR	Schizophrenia 100	12.52
19 SQLS	Wilkinson et al., [79] (revision 4)	Martin & Allen,^43^	UK population (NR)	Community (71%), day centre (13%), and inpatient (16%)	Cross sectional design	40.7 (11.43)	74	NR	Schizophrenia 100	24.64
19 SQLS	Wilkinson et al., [79] (Chinese version, revision 4)	Su et al., [68]	Tiawan (not specified)	Inpatient	NR suggestive of Cross sectional,	49.2 (7.8)	66	NR	Schizophrenia 100	26.6
20 QOL-BD	Development study	Michalak & Murray, [47]	USA and Canada (English)	Inpatient (58.6%) and outpatients	Longitudinal	41.03 (13.8)	31.5	NR	Bipolar type 1 and 2 100	NR
21 Q-LES-Q-18	Development study based on Endicott et al., [18]	Ritsner et al., [61]	Israel (NR)	Inpatient (71.8%) and outpatients	Repeated measures	38.5 (10.3)	79.8	Jewish or Arab Israelis	Schizophrenia and Schizoaffective disorders 100	13.7
22 Henrich’s Quality of Life Scale	Henrich’s et al., [29]	Simon-Abbadi et al., [64]	France (French)	Inpatient	NR suggestive of Repeated measures	31.5 (8.5)	63.3	NR	Schizophrenia 100	8.5

‡ Age ranges reported as mean (SD) data unavailable

Key: NR = not reported

### Risk of bias

Table 3 reports the psychometric properties from each study as scored against the COSMIN risk of bias assessment [48]. Criterion validity is not included in any tables, as no further results were available for this property. This reflects the lack of a recognised gold standard QoL instrument for this population to serve as a comparator (Blome & Augustine, [8]; [10, 16]).

Risk of bias varied by psychometric property. Cross-cultural validity was rated “inadequate” across all reporting studies due to the lack of qualitative and quantitative methods supporting translation and adaptation. Internal consistency was generally rated “very good”, although some studies were assessed as lower quality where Cronbach’s Alpha was not calculated. Construct validity received “inadequate” and “doubtful” ratings in studies that lacked predefined hypotheses or used inappropriate statistical analysis. Structural validity ratings were affected by insufficient sample sizes. COSMIN recommends a sample of at least five times the number of items in the scale for sufficient structural validity. Content validity assessments lacked an appropriate level of detail. Common limitations included the absence of saturation, missing interview guides and unclear transcription procedures. Test-retest reliability was rated lower when studies did not report the time interval between administrations, assess sample stability or provide intra-class correlation (ICC) values. These methodological limitations contribute to the reduced certainty of evidence across several psychometric domains.

**Table 3:** Quality of studies: risk of bias assessment

OMI	Reference	Content validity	Structural validity	Internal consistency	Cross cultural validity	Reliability	Measurement error	Construct validity		Responsiveness
								Convergent	Discriminant	
1 LQoLI-BREF	Anderson et al., [2]							A	A	I
2 LQoLI Full	Russo et al., [63]		A	VG				VG	VG	VG
2 LQoLI Full	Lehman, [38]	I		VG		A		VG	VG	
2 LQoLI Full	Goodwin & Madell, [26]							I	A	
3 Q-LES-Q	Bishop et al., [7]		I	VG						
3 Q-LES-Q	Ritsner et al., [62] (10 items removed)			VG	I	A		A	VG	
4 Q-LES-Q SF	Pitkanen et al., [58]		VG	VG				A	A	
5 LQOLP	Ritsner et al., [62] (only 27 items considered)			VG	I	A		A	VG	
5 LQOLP	Kaiser et al., [32]		I	VG				A	A	
5 LQOLP	Gaite et al., [24]			VG	I	VG				
5 LQOLP	Hansson et al., [28]			VG	I	I				
6 S-QOL-18	Boyer et al., [9]		VG	VG				VG	VG	A
7 SQOL-41	Auquier et al., [5]	A	A	VG		I		VG	VG	A
7 SQOL-41	Chou et al., [12]			VG	I	VG		A	A	
8 QLI	Angstman et al., [3]									I
9 QLIS	Franz et al., [22]	A		VG		A				
9 QLIS	Franz et al., [23]							VG		
10 How Are You? Scale	Katsavdakis et al., [34]	D							A	A
11 QLS	Nair et al., [49]	I		VG					D	I
12 SF-36	Newnham et al., [50] (only subscales related to mental health)								A	D
12 Sf-36	Pukrop et al., [60]			VG				A	A	D
12 Sf-36	Nishiyama et al., [52]			VG				A	A	
12 Sf-36	Su et al., [67]		I			A	A	A	D	
12 Sf-36	Tunis et al., [71]		VG	VG				A	A	D
13 WHO-QOL-BREF	Su et al., [67]		I			A	A	A	D	
13 WHOQOL-BREF	Oliveira et al., [54]		VG	VG				A		
13 WHOQOL-BREF	Norholm & Bech, [53]			I					D	
13 WHOQOL-BREF	Chan et al., [11]							A	A	
13 WHOQOL-BREF	Willige et al., [72]							A		D
14 MSQOL	Pukrop et al., [60]			VG				A	A	D
15 EQ-5D	Pikanen et al., [58]		VG	VG				A	D	
15 EQ-5D	Willige et al., [72]							A		D
16 MHQOL	Ebadi & Rezaiye, [15]	D	VG	VG	I	A	A			
17 SF-12	Soysal Gunduz et al., [65]		VG	VG				D	D	
18 ReQoL	Keetharuth et al., [35]	A	VG	VG		A		VG	VG	VG
19 SQLS	Kaneda et al., [33]		I	VG	I			VG		
19 SQLS	Kuo et al., [36]			VG		A		VG		A
19 SQLS	Kuo et al., [37]		I				A	A		
19 SQLS	Martin & Allen, 43		I	VG					A	
19 SQLS	Su et al., [68]		I					VG		
20 QOL-BD	Michalak & Murray, [47]	VG	I	VG		D		A		
21 Q-LES-Q 18	Ritsner et al., [61]		VG	VG		A		A	A	
22 Henrichs QoL Scale	Simon-Abbadi et al., [64]		I	VG		I	D	A		

Key: COSMIN risk of bias ratings are rated on a scale of Inadequate (I), Doubtful (D), Adequate (A) and Very Good (VG).

## Summarising the results

A total of 22 OMIs were included in this review (Table 4); none covered all eight psychometric properties. Further numerical details of psychometric properties are available in the supplementary materials (Supplementary Table 2). It is recommended that data be extracted by one reviewer and checked by a second [48]. A second reviewer independently assessed 13 of the 38 included studies (33%) for risk of bias. In two cases, disagreements relating to construct validity were resolved through consultation of the COSMIN manual and a consensus discussion. Some of the outcome measures (e.g., LQOLP, QLI, QLS, EQ-5D) are formative. This indicates that certain psychometric properties, such as structural validity and internal consistency, may not be appropriate for these instruments, as their domains are conceptually independent. Findings should be interpreted with this understanding.

Content validity was reported for eight OMIs (Supplementary Table 3). The ReQoL reports content validity from patients in a community mental health team (CMHT), so the results may not be relevant to an inpatient population. This study was included because other measurement properties were tested with an inpatient sample, and the review aims to highlight potential areas for the development of OMIs for this population. The SF-36, and EQ-5D were not explicitly developed for mental health or inpatient purposes; they were designed as generic instruments for measuring health related QoL and evaluating medical outcomes and healthcare interventions. Michalak and Murray, [47] employed appropriate methods to assess the relevance and comprehensibility of the QOLBD among staff and patients. Furthermore, they conducted a pilot study. This indicates that it was adequately assessed according to COSMIN.

Of the OMIs identified, three demonstrated sufficient evidence across six measurement properties: the SQOL-41 (OMI 7), SF-36 (OMI 12), and ReQoL (OMI 18). The SQOL-41 was evaluated across two studies and demonstrated sufficient evidence of content validity, structural validity, internal consistency, construct validity, and test-retest reliability. The translation process of both French and English versions of the scale was described; however, insufficient information was provided about group comparisons to rate the scale. Measurement error was not reported, which impacted the certainty of the evidence.

The SF-36 was evaluated in five studies and demonstrated sufficient evidence of structural validity and internal consistency. However, there were differences in internal consistency scores within and across studies. Nishiyama et al., [52] reported inconsistent results (0.26–0.91), which were due to the differing levels of cognitive functioning between the study populations. Test-retest reliability, measurement error, construct validity and responsiveness were sufficient. However, there were some differences between studies for responsiveness. For instance, one author [50] used RCI (1.96**) to measure responsiveness instead of effect size. Content validity and cross-cultural validity were not reported, impacting the strength of the OMI.

The ReQoL reports seven of eight psychometric properties. There was sufficient evidence demonstrated for five measurement properties including structural validity, internal consistency, test-retest reliability, construct validity and responsiveness. Content validity was conducted in an outpatient setting and it was deemed important to report on to highlight the gap for inpatient settings. The authors conducted a translatability assessment and a differential item functioning (DIF) analysis; however, there was insufficient evidence to demonstrate these properties. Measurement error was not reported.

Two OMIs reported two or fewer sufficient psychometric properties, indicating that these OMIs may be the least robust QoL measures in inpatient settings. The QLI demonstrated sufficient evidence for responsiveness, and the How Are You? Scale reported sufficient evidence for construct validity and responsiveness.

Data limitations prevented a meta-analysis. In the case of outcome measures with potential for meta-analysis, they lacked an appropriate data range, such as unidimensionality and content validity, to produce meaningful results.

Overall, a small subset of OMIs, including S-QOL-41, ReQoL, and SF-36 demonstrated consistently strong evidence across multiple psychometric properties. However, common limitations across OMIs included poor or absent content and cross-cultural validity and a lack of predefined hypotheses for construct validity and responsiveness.

**Table 4:** Results table: measurement properties

Instrument name	Reference	Sample size (*N*)	Measurement model	Content validity	Structural validity (rating)	Internal consistency (rating)	Cross-cultural validity (rating)	Reliability (rating)	Measurement error (rating)	Construct validity (rating) (rating)	Responsiveness (rating)
1 LQoLI-BREF	Anderson et al., [2]	CV R, *N* = 47	reflective	NR	NR	NR	NR	NR	NR	CV (+) DV (+)	(-)
2 LQoLI Full	Russo et al., [63]	SV, IC, CV and R, *N* = 981	reflective	(+)	(+)	(+)	NR		NR	CV: (+) DV: (+)	(+)
	Lehman, [38]	CV, *N* = 469 Re, *N* = 45 SV, *N* = 99				(-)	NR	(-)	NR	CV (+) DV(+)	
	Goodwin & Madell, [26]	CV, *N* = 45					NR		NR	CV (+) DV (+)	
3 Q-LES-Q	Bishop et al., [7]	SV, IC, *N* = 151	reflective	NR	(?)	(?)					NR
	Ritsner et al., [62] (10 items removed)	IC, *N* = 199 (inpatient and outpatient sample) CV, *N* = 374 (patient and comparison group) Re, *N* = 20 (inpatient)		NR		(?)	(?)	(-)	NR	CV (+) DV(+)	NR
4 Q-LES-Q SF	Pitkanen et al., [58]	CV, *N* = 280 SV, *N* = 184 IC, *N* = 190	reflective	NR	(+)	(+)	NR	NR	NR	CV: (+) DV: (+)	NR
5 LQOLP	Ritsner et al., [62] (only 27 items considered)	IC, *N* = 374 CCV *N* = 10 Re, *N* = 20 CV, Patients *N* = 199 Professionals *N* = 175	formative	NR		(-)^a^	(?)	(-)	NR	CV (+) DV(+)	NR
	Kaiser et al., [32]	SV, IC, CV, *N* = 440		NR	(-)	(?)			NR	CV(+) DV (+)	NR
	Gaite et al., [24]	IC, *N* = 404 Re, *N* = 294		NR		(?)	(?)	(+)	NR		NR
	Hansson et al., [28]	SV, *N* = 29 Re, *N* = 21		NR		(?)	(?)	(?)	NR		NR
6 S-QOL-18	Boyer et al., [9]	SV, IC, *N* = 517 Re, *N* = 72 CV, *N* = 302 R, *N* = 28	reflective	NR	(+)	(+)	NR	(+)	NR	CV (+) DV (+)	(+)
7 SQOL-41	Auquier et al., [5]	SV, IC, CV, *N* = 207 Re, *N* = 53 R, *N* = 46	reflective	(+)	(+)	(+)		(+)	NR	CV (+) DV (+)	(+)
	Chou et al., [12]	IC, Re and CV, *N* = 41				(+)	(?)		NR	CV (+) DV (+)	
8 QLI	Angstman et al., [3]	R, *N* = 56	formative	NR	NR	NR	NR	NR	NR	NR	(+)
9 QLiS	Franz et al., [22]	ConV *N* = 268 Re, *N* = 49 (outpatients) IC, *N* = 135	reflective	(+)	NR	(-)	NR	(+)	NR		NR
	Franz et al., [23]	CV, *N* = 135			NR		NR		NR	CV (+)	NR
10 How Are You? Scale	Katsavdakis et al., [34] (study 2)	CV, *N* = 117 R, *N* = 43	reflective	(+)	NR	NR	NR	NR	NR	DV (+)	(+)
11 QLS	Nair et al., [49]	IC, CV and R, *N* = 50	formative	(+)	NR	(-)a	NR	NR	NR	DV (+)	(-)
12 Sf-36	Newnham et al., [50] (only subscales related to mental health)	CV, R, *N* = 1830	reflective	NR			NR			DV (+)	(+)
	Pukrop et al., [60]	SV, CV, R, *N* = 205		NR		(+)	NR			CV(+) DV(+)	(+)
	Nishiyama et al., [52]	IC, CV, *N* = 137		NR		(-)	NR			CV (+) DV (+)	
	Su et al., [67]	SV, Re, ME CV, *N* = 100		NR	(+)		NR	(+)	(+)	CV (+) DV (+)	
	Tunis et al., [71]	SV, IC, R, *N* = 1155 CV, patient sample *N* = 1155 general population *N* = 2474		NR	(+)	(+)	NR			CV (+) DV(+)	(+)
13 WHOQOL-BREF	Su et al., [67]	SV, Re, ME, CV, *N* = 100	reflective	NR	(+)		NR	(+)	(+)	CV (+) DV(+)	NR
	Oliveira et al., [54]	SV, *N* = 403 IC, *N* = 191 CV, *N* = 403		NR	(+)	(-)	NR			CV (+)	NR
	Norholm & Bech, [53]	IC, CV, *N* = 38		NR		(?)	NR			DV (+)	NR
	Chan et al., [11]	CV, *N* = 207		NR			NR			CV (+) DV (-)	NR
	Willige et al., [72]	R, *N* = 63		NR			NR			CV (+)	(+)
14 MSQOL	Pukrop et al., [60]	IC, CV, R, *N* = 205		NR	NR	(?)	NR	NR	NR	CV (+) DV (+)	(+)
15 EQ-5D	Pikanen et al., [58]	SV, *N* = 282 IC, *N* = 281 CV, *N* = 280	formative	(+)	(-)a	(-)a	NR	NR	NR	(+)	
	Willige et al., [72]	CV, R, *N* = 63					NR	NR	NR	(+)	(+)
16 MHQOL	Ebadi & Rezaiye, [15]	SV, *N* = 150 IC, Re, ME, *N* = 60	reflective	(+)	(+)	(+)	(?)	(+)	(+)	NR	NR
17 SF-12	Soysal Gunduz et al., [65]	SV, IC, CV, *N* = 136	reflective	NR	(+)	(+)	NR	NR	NR	CV (?) DV (+)	NR
18 ReQoL	Keetharuth et al., [35]	Study 1 = 2262 study 2 = 4266 R, *N* = 953 Re, *N* = 2800 CV, *N* = 4266 SV, *N* = 4266	reflective	(?)	(+)	(+)	(?)	(+)	NR	CV (+) DV(+)	(+)
19 SQLS	Kaneda et al., [33]	SV, IC, CCV, CV, *N* = 55	reflective	NR	(+)	(+)	(?)			CV (+)	
	Kuo et al., [36]	IC, *N* = 97 Re, *N* = 83 CV, *N* = 97 R, *N* = 14		NR		(+)	NR	(+)	(+)	CV (+)	(?)
	Kuo et al., [37]	SV, CV, *N* = 100		NR	(+)		NR			CV(+)	
	Martin & Allen, 43	SV, IC, CV, *N* = 100		NR	(-)	(+)	NR			CV (+)	
	Su et al., [68]	SV, CV, *N* = 100		NR	(+)		NR			CV (+)	
20 QOL-BD	Michalak & Murray, [47]	SV, IC, *N* = 224 Re, CV, *N* = 93	reflective	(+)	(+)	(+)	NR	(-)	NR	CV (+)	NR
21 Q-LES-Q-18	Ritsner et al., [61]	SV, IC, CV, *N* = 472 outpatients’ Re, *N* = 33	reflective	NR	(+)	(+)	NR	(+)	NR	CV (+) DV (+)	NR
22 Henrich’s Quality of Life Scale	Simon-Abbadi et al., [64]	IC, SV, Re, CV, *N* = 60	reflective	NR	(-)	(-)	NR	(+)	(?)	(+)	NR

Key: CV= construct validity, R= responsiveness, SV= structural validity, IC= internal consistency Re= reliability, CCV= cross-cultural validity, ConV= content validity, ME= measurement error

a May not be relevant for a formative measure

### Quality of the evidence (QOE)

The GRADE approach [27] was used to assess the overall QOE for each measurement property across the included studies, as presented in Table 5. Overall, a small subset of OMIs, including S-QOL-41, ReQoL, SF-36, and MHQOL demonstrated sufficient evidence across multiple measurement properties. There were common limitations across OMIs, including poor or absent content and cross-cultural validity, and a lack of predefined hypotheses for construct validity and responsiveness.

OMI 7, the SQOL-41, reported sufficient evidence for content validity, structural validity, internal consistency, reliability, construct validity and responsiveness. Cross-cultural validity was mentioned, but there was indeterminate evidence reported. The “low” QOE ratings for structural validity, responsiveness, and reliability were impacted by sample size. Furthermore, the reliability results were rated as “very low” due to the “inadequate” risk of bias rating. Overall, this scale shows promising evidence for use in an adult psychiatric inpatient setting. However, this scale was designed as a Schizophrenic specific scale and might not be useful across multiple mental health diagnoses seen in an adult inpatient population. Furthermore, the study was conducted with a partial inpatient population, which is reflected in the QOE score.

OMI 12, the SF-36, reported sufficient, “moderate” QOE for structural validity, reliability, measurement error, and construct validity. There is sufficient “low” QOE for responsiveness, and there is inconsistent “low” QOE reported for internal consistency. This is due to multiple populations and multiple studies, which have a low risk of bias score.

OMI 18, the ReQoL demonstrates sufficient evidence of “moderate” quality for structural validity, internal consistency, construct validity and responsiveness. There was sufficient, “low” QOE of reliability due to a partial inpatient population. There was “very low” quality evidence for content validity due to the outpatient sample.

OMI 16, MHQOL demonstrated sufficient high-quality evidence for structural validity, sufficient moderate QOE for internal consistency and “low” QOE for content validity, reliability, and measurement error, due to low sample size. Cross-cultural validity was rated as “indeterminate” and “very low” quality due to a lack of information.

The QLS, ED-5Q and LQoLP are formative measures and measurement properties such as structural validity and internal consistency may not be appropriate for these measures.

The results indicate that no OMI validated in an adult inpatient psychiatric setting covers all measurement properties. Some outcome measures demonstrate sufficient good quality evidence of measurement properties, but without adequate content validity conducted in an inpatient setting, it is difficult to know how meaningful these OMIs would be for adult psychiatric inpatients. In summary, none of the OMIs reviewed cover all the required measurement properties set out by COSMIN [48].

**Table 5:** A summary of the quality of evidence (QOE)

Measurement property		OMI																					
		1 LQoLI BREF	2 LQoLI	3^a^ Q-LES-Q	4 Q-LES-Q SF	5^b^ LQoLP	6 SQOL-18	7^c^ SQOL-41	8 QLI	9 QLIS	10 HAY?	11 QLS	12^d^ SF-36	13 WHOQOL-BREF	14 MSQOL	15 EQ-5D	16 MHQoL	17 SF-12	18 ReQoL	19 SQLS	20 QoLBD	21 Q-LES-Q18	22^e^ HQOLS
Content Validity	Rating		+					+		+	+	+				+	+		?^f^		+		
	QOE		VL					M		M	VL	VL				VL	L		VL		M		
Structural validity	Rating		+	?	+	-^g^	+	+					+	+		-g	+	+	+	+/-	+	+	-*
	QOE		M	VL	H	VL	M	L					M	M		H	H	M	M	VL	VL	M	VL
Internal consistency	rating		+/-	?	+	-/? g	+	+		-		-g	+/-	-/?	?	-g	+	+	+	+	+	+	-*
	QOE		M	M	H	M	M	M		M		M	L	L	H	H	M	M	M	M	M	M	L
Cross-cultural validity	Rating					?		?									?						
	QOE					VL		VL									VL						
Reliability	rating		-			+/?	+	+		+f			+	+			+		+	+	-	+f	+
	QOE		VL			M	L	VL		VL			M	M			L		L	L	VL	VL	VL
Measurement Error	Rating												+	+			+			+			?
	QOE												M	M			L			VL			VL
Construct Validity	rating	+	+		+	+	+	+		+	+	+	+	+/-	+	+		?/+	+	+	+	+	+
	QOE	VL	M		M	M	M	M		M	M	VL	M	L	M	M		VL	M	M	L	L	VL
Responsiveness	Rating	-	+				+	+	+		+	-	+	+	+	+			+	?			
	QOE	VL	M				VL	L	VL		L	VL	L	VL	L	L			M	VL			

^a^ one study has 10 items removed and can’t be pooled

^b^ one study only contained 27 items and so was not included in the summary of results

^c^ SQOL41 and SQOL41 Chinese version

^d^ combined score includes multiple language versions, one study excluded from summary as it only includes 14 items

^e^ one study referenced a different language version, but same items. All items remain the same, but the number of domains varies across studies

^f^ studies that use outpatients for these domains

^g^ Formative measure, internal consistency and structural validity may not be appropriate for these measures

### Reliable change index (RCI)

RCIs have been calculated for each study that reported the means and standard deviations (SD) for a patient population, along with Cronbach’s Alpha, and are presented in Table 6. Some studies did not report the total QoL score and instead reported domain scores; in these cases, the RCI for each domain was calculated. As shown in Table 6, RCI values vary widely from small (e.g., 0.44) to large (e.g., > 30), reflecting differences in scaling, measurement domains, and variance. Those with the lowest RCI (< 6), those requiring lower change for significance, include the Q-LES-Q variants, ReQoL-20, and QOL-BD, while the highest RCI (> 6) include SQLS, SQOL-41, SF-12, and Henrich’s QOL scale. Most instruments reported high internal consistency (α > 0.8), which supports RCI reliability estimates. Despite mixed inpatient/outpatient samples, Cronbach’s α values were consistent, suggesting internal reliability was not a limiting factor, but scaling and item heterogeneity may be.

**Table 6:** Reliable change index calculations

Instrument name	Citation	Sample *N*	Domain	Mean (SD)	Cronbach’s alpha	RCI (CI)
OMI 3 Q-LES-Q	Ritsner et al., [62]	199 (inpatient)	Health	45.4 (10.8)	0.91	9.98 (8.32–9.98)
			Work	30.8 (10.9)	0.9	9.55 (8.77–10.52)
			Leisure time activities	21.1 (6.9)	0.84	7.65 (6.65–8.87)
			Social relationships	37.6 (10.4)	0.86	10.79 (9.33–12.45)
			Life satisfaction	3.5 (1.1)	–	–
			Perceived QOL index	3.5 (0.8)	0.94	0.54 (0.68–0.68)
			Subjective feelings	52.1 (13.2)	0.93	9.68 (8.8–11)
			Household duties	30.1 (14.5)	0.9	12.71 (11.39–14.02)
			General activities	47.6 (11.5)	0.95	7.13 (6.2–8.06)
			Satisfaction with medication	3.7 (1.1)	–	−
OMI 5 LQoLP	Gaite et al., [24]	404 (in and outpatients)	Life Satisfaction Score average score	4.67 (0.76)	0.87	0.76 (1 to 1)
			Global well being	4.37 (1.34)	0.83	1.34 (1–1)
OMI 6 S-QOL-18	Boyer et al., [9]	517 (in and out patients)	Total score	56.4 (18.8)	0.88	18.05 (17.28–19.2)
OMI 7 SQOL-41	Auquier et al., [5]	207 (in and out)	Index score	55 (18.3)	0.94	12.42 (11.54–13.58)
OMI 9 QLIS	Franz et al., [22]	135 (in and outpatient)	Social contacts	4.47 (2.24)	0.63	3.78 (3.37–5.06)
			Appreciation by others	6.37 (2.13)	0.70	3.23 (3.04–3.04)
			Relationship to family	6.15 (2.96)	0.71	4.42 (4.48–4.48)
			Appraisal of pharmacotherapy	5.97 (2.11)	0.71	3.15(2.99–2.99)
			Appraisal of psychopathological symptoms	5.97 (2.11)	0.82	2.82 (2.35–3.53)
			Cognitive functioning	5.49 (2.31)	0.79	2.93 (2.54–3.81
			Abilities to manage daily living	6.42 (2.43)	0.74	3.43 (2.93–2.24)
			Appraisal of accommodation/housing	6.14 (2.17)	0.73	3.13 (2.88–2.88)
			Financial situation	4.72 (2.65)	0.77	3.52 (2.66–3.99)
			Leading a normal life	4.56 (2.6)	0.70	3.95 (3.04–4.55)
			Confidence	5.74 (2.28)	0.74	3.22 (2.83–4.24)
			Global life satisfaction	4.64 (2.75)	0.88	2.64 (1.92–2.88)
OMI 17 SF-12	Soysal Gundaz et al., [65]	55 (inpatient)	Psychosocial	41.9 (20.4)	0.93	14.96 (12.47–18.33)
			Motivation/energy	48.1 (18.2)	0.73	26.21 (21.6-31.69)
			Symptoms/side effects	32.5 (17.6)	0.80	21.82 (18.59–27.27)
OMI 18 ReQoL	Keetharuth et al., [35]	4037 (mixed pop)	ReQoL 10	21.99 (10.26)	0.92	8.31 (7.84–8.62)
			ReQoL 20	21.63 (9.97)	0.96	5.53 (5.54–5.54)
OMI 19 SQLS	Kaneda et al., [33]	55 (inpatient)	Psychosocial	41.9 (20.4)	0.93	14.96 (12.47–18.33)
			Motivation/energy	48.1 (18.2)	0.73	26.21 (21.6-31.69)
			Symptoms and side-effects	32.5 (17.6)	0.80	21.82 (18.59–27.27)
	Kuo et al., [36]	83 (in and outpatients)	Motivation/energy	48.1 (18.2)	0.73	26.21 (21.6-31.69)
			Symptoms and side-effects	32.5 (17.6)	0.80	21.82 (18.59–27.27)
	Martin & Allen, 43	100 (mixed populations)	Psychosocial feelings	38.27 (23.29)	0.96	12.91 (11.09–14.97)
			Cognition and vitality	40.18 (18.17)	0.82	21.37 (18.82–24.7)
			Total score	38.91 (20.99)	0.96	31.37 (18.82–24.7)
OMI 20 QOL-BD	Michalak & Murray, [47]	225 (In and outpatients)	Physical	11.77 (4.01)	0.79	5.09 (5.08–5.08)
			Sleep	11.42 (4.12)	0.83	4.71 (4.57–5.71)
			Mood	12.98 (3.98)	0.90	3.49 (3,51-3.51)
			Cognition	12.78 (4.22)	0.91	3.51 (3.33–4.16)
			Leisure	13.11 (4.16)	0.91	3.46 (3.33–4.16)
			Social	14.31 (4.00)	0.88	3.84 (3.84–3.84)
			Spirituality	13.05 (4.07)	0.93	2.98 (2.93–2.93)
			Finances	12.67 (4.64)	0.88	4.46 (3.84–4.8)
			Household	12.96 (4.11)	0.91	3.42 (3.33–4.16)
			Self-esteem	13.82 (3.70)	0.88	3.55 (2.88–3.84)
			Independence	15.78 (3.23)	0.81	3.9 (3.62–4.83)
			Identity	13.68 (4.14)	0.90	3.63 (3.51–4.38)
			Work	15.20 (3.52)	0.89	4.14 (3.53–4.7)
			Education	13.92 (4.96)	0.95	3.07 (3.1–3.1)
OMI 21 Q-LES-Q-18	Ritsner et al., [61]	339 (in and outpatients)	General QOL index	3.4 (0.8)	0.96	0.44 (0.55–0.55)
OMI 22 Henrich’s Quality of Life Scale	Simon-Abbadi et al., [64]	60 (inpatients)	Mean global score HQLS	41.3 (16.6)	0.9	14.55 (12.27–17.53)

## Discussion

The current review aimed to evaluate the psychometric properties of available QoL measures used in psychiatric inpatient settings. Previous reviews have evaluated specific psychometric properties and reported similar results, including structural validity of the SQLS and SQoLS-R4 (Zuniga Le-Bert et al., 82), Convergent validity and responsiveness for the EQ-5D and SF-36 [56], and the S-QOL-18 and QLIS and Q-LES-Q 18 (Azaiez et al.,[6]). Krugten et al., [74] aimed to assess the characteristics and dimensions of available QoL OMIs used in mental health settings but did not evaluate psychometric properties.

None of the QoL measures included in the review report high quality of evidence for every psychometric property defined by COSMIN. The ReQoL [35] lacked content validity in an inpatient sample, raising concerns about the measure’s use within this setting and the relevance of the items to adult psychiatric inpatients. By comparison, the MHQOL [73] shows promising evidence for use in psychiatric inpatient settings.

### Implications and future research

This review highlights the range of available OMIs with some psychometric properties tested in inpatient populations. Further research should explore content validity in inpatient samples, particularly the ReQoL, a general mental health QoL measure with strong reliability, commonly used in practice within inpatient settings in the United Kingdom. A specific inpatient version of the measure could be developed based on content validity and subsequently retested across various indices. Additional research should be conducted using the MHQOL to gain insights into construct validity and responsiveness for inpatient populations.

Among the studies that reported content validity, most did not provide high-quality evidence. A scale should demonstrate content validity to ensure it is grounded in appropriate theory and evidence, and that its domains are meaningful to its users. Future research should concentrate on developing a scale with sufficient content validity for a psychiatric inpatient population.

COSMIN recommends that if there is insufficient or no content validity, reviewing the other measurement properties becomes redundant. This is because it may not measure the intended construct for the intended population. However, as part of this review, all psychometric properties were evaluated to deepen understanding of the current evidence base. Interpretations can be made regarding OMIs whilst acknowledging content validity as a limitation. It is hoped that, by highlighting the strengths and weaknesses of each scale, future research can build on these areas.

QoL is widely regarded as a multidimensional construct encompassing various life domains. Multidimensional instruments can yield domain-specific scores that identify particular impairments, providing clearer guidance for care planning [42]. COSMIN guidance suggests that QoL domains may be treated as separate outcomes, with analysis of each domain’s psychometric properties [17]. However, due to the limited availability of consistent domain-level data, domains were not analysed as independent outcome measures in this review. Instead, domain names were summarised, and RCIs were calculated at the domain level when sufficient data were available. This method recognised domain-level differences while ensuring consistent synthesis across instruments. Previous reviews have systematically compared the item content of different assessment instruments [30]. Future research could adopt a similar approach to summarise and compare domains across outcome measures, to identify content overlap and prioritise content validity.

RCI scores were calculated for several outcome measures and are presented in the results section. This has implications for understanding change over time using the identified outcome measures. RCIs determine whether observed changes in scores are statistically reliable or due to measurement error, which can guide treatment decisions and inform the use of outcomes in research to understand treatment effectiveness [31].

### Strengths and limitations

This review was conducted in accordance with the COSMIN guidelines. The interpretation of these guidelines relies on the reviewer’s expertise and judgment, presenting subjectivity biases [45]. Due to time and resources, only a portion of the full text screening and quality assessment was conducted by a second reviewer. Transparency of reporting was maintained, further minimising the risk of bias, and the review was pre-registered.

Forward citation searching was not noted in the pre-registered protocol. Although not originally planned, this step enhanced the comprehensiveness of the review and enabled the inclusion of literature that reflects the current direction of the evidence base.

Minor updates were made to the inclusion and exclusion criteria before conducting the formal search strategy. This involved clarifying that only studies reporting and evaluating psychometric properties, such as validation studies, should be included. Future studies could examine interventional studies and extract data on the responsiveness of scales. This was unnecessary at this stage of the review process, as the focus was on understanding a breadth of psychometric properties, not solely responsiveness.

Some studies included in the review contained mixed samples, which have the potential to introduce bias which was considered when rating the quality of evidence. A subgroup analysis [14] could be used to further understand the results for an inpatient population. However, it was deemed unnecessary to meet the aims of the current review. Including a mixed population enabled breadth and depth in literature coverage, including aspects of construct validity that might not have been covered otherwise. This approach enabled the presentation of knowledge gaps across a range of OMIs that may be of value in inpatient settings.

In the search strategy, the Health of the Nation Outcome (HONOS) [80] was included, as it was initially considered a QoL outcome measure. This is a potential limitation of the search strategy, which yielded redundant results, and so future replications might consider excluding HONOS from the search strategy.

Results from multiple studies assessing the same outcome measure were combined using the GRADE approach, which included different language versions. These differences might influence the interpretation of scores and may be considered a limitation.

## Conclusion

Given that no outcome measures were identified that encompassed all COSMIN measurement properties, clinicians should approach the identified outcome measures with caution. Most measures demonstrated good internal consistency and construct validity, indicating some reliability and validity in this setting. Until a measure has been thoroughly tested in an inpatient setting, it can be inferred that there is currently no gold standard, although several of the reviewed measures have adequate psychometric properties across multiple domains.

## Supplementary Information

Below is the link to the electronic supplementary material.

Supplementary Material 1

Supplementary Material 2

## Data Availability

No datasets were generated or analysed during the current study.

## Appendix A

Full search strategy.

**Table:**

Number	Search term
1	“psy* hospital*”
2	“psy* unit*
3	“psy* inpatient*”
4	“psy* ward*”
6	“psy* facility*”
6	“psy* institution*”
7	“mental* ward*”
8	mental* hospital*”
9	“mental* unit*”
10 11 12 13 14 15 16 17 18 19 20 21 22 23 24 25 26 27 28 29 30 31 32 33 34 35 36 37 38 39	“mental* inpatient*” “mental* facility*” “mental* institution*” “psychiatric care” “psychiatric services” “psychiatric treatment” “psychiatric rehabilitation” “mental health rehabilitation” “psychiatric admission*” “psychiatric hospitalization” “psychiatric readmission*” “psychiatric discharge*” “Tier 4 mental health” “Tier 4 service*” “highly specialized mental health” “specialist mental health inpatient*” “tertiary mental health care” “tertiary psychiatric care” “specialist psychiatric unit*” “high dependency mental health unit*” “long-stay psychiatric hospital*” “chronic psychiatric inpatient care” “specialist inpatient mental health service*” “hospital-based mental health care” Psychometric* “factor analysis” Valid* Reliab* “responsiveness” “internal consistency”
40	Measure*
41	instrument
42	scale
43	outcome
44 45	Index PROM
46	“quality of life”
47	SQOL
48	QOL
49	ReQol
50	“EuroQol-5
51	q-les-q
52	EQ-5D
53	EuroQol-5 Dimension
54	MHQoL
55	SQLS
56	BQOL
57	QLDS
58	SF-36
59	Short Form Health Survey-36
60	HoNOS
61	SF-12
62	SF-6D
63	WHOQOL
64	EUROHIS-QOL
65	AQoL
66	“15 dimensional”
67	QLI
68	QOLS
69	CDC HRQOL-4
70	PWI-A
71	“Personal well-being index-adult”
72	QOL5

### Scopus search

( TITLE-ABS-KEY ( psychometric* OR "factor analysis" OR valid* OR reliab* OR responsiveness OR "internal consistency" OR measure* OR outcome* OR instrument OR scale OR index OR prom ) AND TITLE-ABS-KEY ( "quality of life" OR qol OR sqol OR reqol OR "EuroQOL-5" OR q-les-q OR eq-5d OR mhqol OR sqls OR bqol OR qlds OR sf-36 OR "short form health survey- 36" OR honos OR sf-12 OR sf-6d OR whoqol OR eurohis-qol OR aqol OR "15 dimensional" OR qli OR qols OR "CDC HRQOL-4" OR pwi-a OR "personal well-being index-adult" OR qol5 ) AND TITLE-ABS-KEY ( "psy* hospital*" OR "psy* unit*" OR "psy* inpatient*" OR "psy* ward*" OR "psy* facility*" OR "psy* institution*" OR "mental* ward*" OR "mental* hospital*" OR "mental* unit*" OR "mental* inpatient*" OR "mental* facility*" OR "mental* institution*" OR "psychiatric care" OR "psychiatric services" OR "psychiatric treatment" OR "psychiatric rehabilitation" OR "mental health rehabilitation" OR "psychiatric admission*" OR "psychiatric hospitalization" OR "psychiatric readmission*" OR "psychiatric discharge*" OR "Tier 4 mental health" OR "Tier 4 service*" OR "highly specialized mental health" OR "specialist mental health inpatient*" OR "tertiary mental health care" OR "tertiary psychiatric care" OR "specialist psychiatric unit*" OR "high dependency mental health unit*" OR "long-stay psychiatric hospital*" OR "chronic psychiatric inpatient care" OR "specialist inpatient mental health service*" OR "hospital-based mental health care" ) ) AND ( LIMIT-TO ( LANGUAGE , "English" ) )

### PsycInfo search

1. (psychometric* or “factor analysis” or valid* or reliab* or responsiveness or “internal consistency” or measure* or outcome* or instrument or scale or index or prom).ab, ti.
2. treatment outcomes/ or psychotherapeutic outcomes/ or patient reported outcome measures/patient reported outcome measures/ or “treatment process and outcome measures”/ or self-report/ or treatment outcomes/rating scales/ or likert scales/.
3. foreign language translation/ or cross cultural test adaptation/ or cross cultural validity/.
4. exp Test Validity/ or exp Psychometrics/ or exp Test Reliability/.

5. factor structure/ or factor analysis/.
6. exp Construct Validity/ or exp Confirmatory Factor Analysis/.
7. 1 or 2 or 3 or 4 or 5.
8. (“quality of life” or qol or sqol or reqol or “EuroQOL” or q-les-q or eq-5d or mhqol or sqls or bqol or qlds or sf-36 or “short form health survey-36” or honos or “health of nation outcome scale” or sf-12 or sf-6d or whoqol or eurohis-qol or aqol or “15-dimensional” or qli or qols or “CDC HRQOL-4” or pwi-a or “personal well-being index-adult” or qol5).ab, mf, ti.
9. “quality of life”/ or “quality of life measures”/.
10. 7 or 8.
11. (“psy* hospital*” or “psy* unit*” or “psy* inpatient*” or “psy* ward*” or “psy* facility*” or “psy* institution*” or “mental* ward*” or “mental* hospital*” or “mental* unit*” or “mental* inpatient*” or “mental* facility*” or “mental* institution*” or “psychiatric care” or “psychiatric services” or “psychiatric treatment” or “psychiatric rehabilitation” or “mental health rehabilitation” or “psychiatric admission*” or “psychiatric hospitalization” or “psychiatric readmission*” or “psychiatric discharge*” or “Tier-4 mental health” or “Tier-4 service*” or “highly specialized mental health” or “specialist mental health inpatient*” or “tertiary mental health care” or “tertiary psychiatric care” or “specialist psychiatric unit*” or “high dependency mental health unit*” or “long-stay psychiatric hospital*” or “chronic psychiatric inpatient care” or “specialist inpatient mental health service*” or “hospital-based mental health care”).ab, mf, ti.
12. psychiatric hospitalization/ or psychiatric hospital admission/ or psychiatric hospital discharge/ or psychiatric hospital readmission/ or mental health commitment/.
13. psychiatric units/ or psychiatric hospitals/.

14. 10 or 11.
15. 6 and 9 and 12.

### Medline search

1. psychometric* or “factor analysis” or valid* or reliab* or responsiveness or “internal consistency” or measure* or outcome* or instrument or scale or index or prom).ab, ti.
2. Thesaurus search: Psychometrics/.
3. 1 or 2.
4. “quality of life” or qol or sqol or reqol or “EuroQOL” or q-les-q or eq-5d or mhqol or sqls or bqol or qlds or sf-36 or “short form health survey-36” or honos or “health of nation outcome scale” or sf-12 or sf-6d or whoqol or eurohis-qol or aqol or “15-dimensional” or qli or qols or “CDC HRQOL-4” or pwi-a or “personal well-being index-adult” or qol5).ab, ti.
5. Thesaurus search: “quality of life”/ or psychological well-being/.
6. 4 or 5.
7. (“psy* hospital*” or “psy* unit*” or “psy* inpatient*” or “psy* ward*” or “psy* facility*” or “psy* institution*” or “mental* ward*” or “mental* hospital*” or “mental* unit*” or “mental* inpatient*” or “mental* facility*” or “mental* institution*” or “psychiatric care” or “psychiatric services” or “psychiatric treatment” or “psychiatric rehabilitation” or “mental health rehabilitation” or “psychiatric admission*” or “psychiatric hospitalization” or “psychiatric readmission*” or “psychiatric discharge*” or “Tier-4 mental health” or “Tier-4 service*” or “highly specialized mental health” or “specialist mental health inpatient*” or “tertiary mental health care” or “tertiary psychiatric care” or “specialist psychiatric unit*” or “high dependency mental health unit*” or “long-stay psychiatric hospital*” or “chronic psychiatric inpatient care” or “specialist inpatient mental health service*” or “hospital-based mental health care”).ab, ti.
8. Hospitals, Psychiatric/.
9. 7 or 8.
10. 3 and 6 and 9.

### Cinahl search

"psychiatric inpatient*" OR "mental health inpatient*" OR "psychiatric unit*" OR "mental health facility" OR "psychiatric care" OR "mental health services" OR "specialist inpatient mental health" OR “psychiatric hospital” OR “psychiatric unit” OR “psychiatric inpatient” OR “psychiatric ward” OR “psychiatric facility” OR “psychiatric institution” OR “mental health ward” OR “mental health hospital” OR “mental health unit” OR “mental health inpatient” OR “mental health facility” OR “mental health institution” OR “psychiatric care” OR “psychiatric services” OR “psychiatric treatment” OR “psychiatric rehabilitation” OR “mental health rehabilitation” OR “psychiatric admission” OR “psychiatric hospitilization” OR “psychiatric readmission” OR “psychiatric discharge” OR “tier 4 mental health” OR “highly specialised mental health” OR “specialist mental health inpatient” OR “tertiary mental health care” OR “tertiary psychiatric care” OR “specialist psychiatric unit” OR “high dependency mental health unit” OR “long stay psychiatric hospital” OR “chronic psychiatric inpatient care” OR “specialist inpatient mental health service” OR “hospital based mental health care”

AND

"recovering quality of life" OR "reqol" OR "reqol-10" OR "reqol-20" OR qol OR sqol OR reqol OR eurqol OR q-les-q OR eq-5d OR mhqol OR sqls OR bqol OR qlds OR sf-36 OR “short form health survey-36” OR honos OR “health of nation outcome scale” OR sf-12 or sf-6d OR whoqol OR eurohis-qol OR aqol OR 15-dimensional OR qli OR qols OR CDC hrqol-4 OR pwi-a OR “personal wellbeing index adult” OR qol5 OR “health related quality of life”

AND

psychometrics OR validity OR reliability OR “psychometric properties” OR “factor analysis” OR responsiveness OR “internal consistency” OR “outcome measures” OR “outcome assessment” OR “outcome tool” OR “outcome instrument” OR “outcome scale” OR “likert scale” OR PROM OR “patient reported outcome measure”CINAHL headingsQuality of lifePsychometricsPsychiatric inpatients
