# Supplementary material for: Systematic review of the psychometric properties of quality-of-life outcome measures used with adult inpatient psychiatric populations
Source: Qual Life Res. 2026 Apr 1;35(5):127. doi: 10.1007/s11136-026-04241-9 (PMC13043520; doi:10.1007/s11136-026-04241-9)
Supplement: Supplementary file 2 — Supplementary Material 2 [file 11136_2026_4241_MOESM2_ESM.docx]

**Supplementary Tables**

**Table 1**

*Outcome Measurement Instruments Characteristics*

| OMI | OMI | Reference | Items (N) | Administration time (minutes) | Scale Domains (N items) | Response options  (Likert) | Scoring | Recall period | Measure Available(Y/N) | Administration format | Languages  Versions included |
| --- | --- | --- | --- | --- | --- | --- | --- | --- | --- | --- | --- |
| 1 | Lehamn’s Brief Quality of life interview | Anderson et al., 2002 | 74 | 16 | Objective (2), subjective QoL (6) and global life satisfaction score (1) | 1-7 | Average, higher score indicates higher QoL | One year | Y | interviewer administered. (by trained clinicians) | English |
| 2 | LQoLI (full) | Russo et al., 1997 | 158 | 45 | Objective QoL: Living situation (8), frequency of family contacts (1), frequency of social contacts (1), number of leisure activities (1), work (1), frequency of religious activity, finances (2), safety (2), health (3)  Subjective QoL: General life satisfaction (living, family, social, leisure, work, religious, finances, safety, health). | 1-7 | A higher score indicates higher QoL | NR | Y | interviewer administered. (by nursing staff) | English |
|  |  | Lehman, 1988 |  |  |  |  |  |  |  | Interviewer administered (by trained staff) |  |
| 3 | Q-LES-Q | Bishop et al., 1999 | 90/93 (3 optional functioning items | 40-45 | Four of the core subscales assess the global domains of physical health (13 items), subjective feelings (14 items), leisure activities (six items), social relationships (11 items), and general activities, work, household duties, and school/course work. There are single questions addressing overall satisfaction and medication satisfaction. Items on school and coursework have been excluded from Ritsner et al. (2002) | 1-5 | Average score, higher score indicates greater satisfaction | Past week | Y | Self-report | English,  Hebrew |
|  |  | Ritsner et al., 2002 | 83 | 40-45 |  |  |  |  |  |  |  |
| 4 | QLESQ-SF | Pikanen et al. 2011 | 16 | 7 | Fourteen questions assess domains such as social relationships, living or housing situation, feelings and physical health. The last two questions concern medication and overall life satisfaction during the past 7 days. | 1-5 | Total score added, higher score indicates better QoL | Past week | Y | Self-report | Finish |
| 5 | Lancashire Quality of Life Profile (LQoLP) and LQoLP-EU | Ritsner et al. 2002 (27 items) | 27 | 20-25 | 9 domains work and education (7), leisure and participation (8), religion (4), finances (7 items), social relations (6), health 10.  (Life regard index and satisfaction with life scale are additional components to the EU version.) | 7 point | Average scores.  Higher score indicates better QoL | Present | N | Has elements of subjectivity- completed with an interviewer | Hebrew,  Dutch, Danish, English,  Spanish, Italian,  Swedish,  German, |
|  |  | Kaiser et al., 1997 | 100 |  |  |  |  |  |  |  |  |
|  |  | Gaite et al., 2000 | 105 |  |  |  |  |  |  |  |  |
|  |  | Hansson et al., 1998 | 100 |  |  |  |  |  |  |  |  |
| 6 | Schizophrenia Quality of Life Scale (SQOL) -18 Version 1 | Boyer et al., 2010 | 18 | NR | 8 subscales (psychological well-being (3), self-esteem (2), family relationships (2), relationship with friends (2), resilience (3), physical wellbeing (2), autonomy (2), sentimental life (2). Global score of QOL (total) | 1-5 | 0-100 higher score indicating higher QoL | Present | N | Self-report | French |
| 7 | SQOL-41 and Chinese version SQOL-C | Auquier et al., 2003 | 41 | NR | 8 domains, psychological wellbeing (10), self-esteem (6), family relationships (5), relationships with friends (5), resilience (5), physical wellbeing (4), autonomy (4), sentimental life (2) | 1-5 | Means 0-100 with 100 being most favourable | Present | Y | Self-report | English,  Chinese |
|  |  | Chou et al., 2010 |  |  |  |  |  |  |  |  |  |
| 8 | Quality of Life Interview (QLI) | Angstman et al., 2009 | 32 | NR | 16 domains: health, self-esteem, goals-and-values, money, work, play, learning, creativity, helping, love, friends, children, relatives, home, neighbourhood, and community | 1-6 | Average responses, higher score indicates better QoL | NR | N | Self-report | English |
| 9 | Quality of Life in Schizophrenia (QLIS) version 1 | Franz et al., 2012 | 53 (with 2 optional items relating to employment) | NR | 16 subscales social contacts (5 items) appreciation by others (4 items), relationship to my family (3 items), appraisal of pharmacotherapy (6) appraisal of psycho-pathological symptoms (6) cognitive functioning (5) abilities to manage daily living (4) appraisal of accommodation/housing (5) financial situation (4) leading a normal life (3) confidence (4) global life satisfaction (3) | 1-4 | Means (0-10, with zero worst QoL) | NR | N | Self-report | German |
|  |  | Franz et al., 2012 |  |  |  |  |  |  |  |  |  |
| 10 | The How Are You? Scale Version 5 | Katsavdakis et al., 1999 | 55 | 30 | 5 Domains: Your health (6), how you’ve been feeling (20), your daily life (7), your circumstances (6), your relationships (10), overall domain (6) | 0-4 | Means (direction not indicated) | Present | N | Self-report | English |
| 11 | Quality of Life Survey (QLS) | Nair et al., 1996 | 63 | 15-20 | 8 domains: Material quality of life (15 items, 6, 3 and 5), knowledge and education (7 items), human relationships (4), dependency issues (2), inner experience (6), inpatient treatment (9), leisure activities (4), work and vocational rehabilitation (2). | 5 | Mean (direction not indicated) | Preceding month | N | Self-report | NR |
| 12 | Short Form (SF)-36 Health survey | Newnham et al., 2007 (only mental health subscales) | 36 | NR | 8 domains: physical functioning (10) role-physical (4) bodily pain (2) general health (5) vitality (4) social functioning (2) role emotional (3) mental health (5) | Multiple severity scales | Mean, 0-100 with higher score indicating higher QoL | 4 weeks | Y | Self-report  In one study this was administered by trained clinicans | English,  Japanese,  Chinese |
|  |  | Pukrop et al., 2003 |  |  |  |  |  |  |  |  |  |
|  |  | Nishiyama et al., 2009 |  |  |  |  |  |  |  |  |  |
|  |  | Su et al., 2014 |  |  |  |  |  |  |  |  |  |
|  |  | Tunis et al., 1999 |  |  |  |  |  |  |  |  |  |
| 13 | World Health Organisation Quality of Life Assessment WHOQOL-BREF | Su et al., 2014 | 26-28 (2 domestic on the Taiwan version) | A few minutes | 4 domains: physical (5) psychological (5) level of independence (6) social relationships (3) Environment (9) spirituality and religious beliefs, overall quality of life and general health perceptions | 5 point scale | Means, 0-100, higher scores refer to higher QoL | Present | Y | Self report in current studies: can be administered by proxy | Chinese,  Portuguese, Danish, Chinese, Dutch |
|  |  | Oliveira et al., 2016 |  |  |  |  |  |  |  |  |  |
|  |  | Norholm & Bech, 2006 |  |  |  |  |  |  |  |  |  |
|  |  | Chan et al., 2003 |  |  |  |  |  |  |  |  |  |
|  |  | Van de Willige et al., 2005 |  |  |  |  |  |  |  |  |  |
| 14 | Modular System for Quality of Life (MSQOL) | Pukrop et al., 2003 | 55 | NR | Mood rating, sociodemographic module of objective QOL subjective QOL modules: physical health, physical vitality, personal role and social relationships, leisure activities, mental state and emotions, life in general, family, partnership, occupation | 7 point scale | 0-100 higher score indicating higher QoL | 4 weeks | Y | Self-report, clinical psychologist could support in question understanding | English |
| 15 | EuroQOL (EQ)-5D 3L and 5L  (still comparable) | Pikanen et al., 2011 (descriptive scale only 3L) | 5 | NR | Five dimensions  mobility, self-care, daily functioning, pain or discomfort, and anxiety or depression. | 1-3 (3Levels)  1-5 (5Levels) | Scores are coded and a score of 1.00 indicates full health  Or as a visual analogue scale 0-100 (100 being best imaginable) | 7 days | Y | Self-report | Finish, Dutch |
|  |  | Van de Willige et al., 2005 |  |  |  |  |  |  |  |  |  |
| 16 | Mental Health Quality of Life (MHQoL) -7D Questionnaire | Ebadi & Rezaiye, 2025 | 7 | Quick to complete | Self-image, independence, mood, relationships, daily activities, physical health, future, psychological well-being | Level 1- 4 (0-3 score) | Sum of scores 0-21 | Today | Y | Self-reported | Farsi |
| 17 | Short Form (SF)-12 health survey version 2 | Soysal Gunduz et al., 2021 | 12 | NR | 8 domains: General health, physical functioning, Role physical, Bodily pain, Social functioning, Vitality, Mental health, Role-emotional. | 3 point Likert and 5 point for 10 items | NR | Present- 4 weeks | Y- English online and Turkish from author | NR | Turkish |
| 18 | Recovering Quality of Life (ReQoL) 10 and 20 | Keetharuth et al., 2018 | 10 items and 20 items respectively | A few minutes | Generic overall rating of QoL, recovery, and well-being. | 0-4 points  5 levels | summative | Past week | Y (NHS and public research) | Self-administered | English |
| 19 | Schizophrenia Quality of Life Survey (SQLS) | Kaneda et al., 2002 | 30 | NR | Psychosocial (15), motivation/energy (7) and symptoms side effects (8) | 0-4 | Lower score represents better HRQOL | NR | N | Self-administered | Japanese |
|  |  | Kuo et al. 2007 | 33 | NR | two domains, psychosocial (20) and vitality (13) | 0-4 5 point scale | Lower score represents better HRQOL | 7 days | Y- permission needed | Self-report | Chinese |
|  |  | Kuo et al., 2009 |  |  |  |  |  |  |  |  |  |
|  |  | Martin & Allen, 2007 |  |  |  |  |  |  |  |  |  |
|  |  | Su et al., 2017 |  |  |  |  |  |  |  |  |  |
| 20 | Quality of Life in Bipolar Disorder (QOLBD) | Michalak et al., 2010 | 56 | 4 | a revised set of 14 domain subscales were indicated: Physical, sleep, mood, cognition, leisure, social, spirituality, finances, household, self-esteem, independence, identity, work, education | 5 point scale | NR | 7 days | Y | Self-report | English |
| 21 | QLESQ-18 | Ritsner et al., 2005 | 18 | 10-12 | Physical health, subjective feelings, leisure time activities, social relationships, general index. | 5 point scale | Average of all 18 items, higher score is better QoL | Past week | Y | Self-report | English |
| 22 | Heinrich’s Quality of Life Rating Scale | Simon-Abbadi et al., 1999 | 21 | 45 | Domains: interpersonal relations (1-8), instrumental QoL (9-12) intrapsychic foundations (13,17,20,21) Common objects and activities (18-19) | NR | NR | NR | N | Patients interviewed | French |

**Table 2**

*Results*

| Instrument name | Reference | Content validity | Structural validity (rating) | Internal consistency (rating) | Cross-cultural validity (rating) | Reliability (rating) | Measurement error (rating) | Construct validity (rating)  Convergent validity ( *r* )  Discriminant validity  (rating) | Responsiveness (rating) (d) |
| --- | --- | --- | --- | --- | --- | --- | --- | --- | --- |
| 1  LQoLI-BREF | Anderson et al., 2002 | NR | NR | NR | NR | NR | NR | CV: intercorrelations within subjective scales: 0.37- 0.62* Objective scales independent, one significant relationship between social contact and finance: 0.55** (+)  DV: No differences between patient and normative sample except:  living situation: MD= 0.88-1.05**  Family relationships: MD= 0.48*  Family contact: MD=0.35-0.42* (+) | The scale was reported stable across time (6 weeks) effect size not calculated (-) |
| 2  LQoLI Full | Russo et al., 1997 | (+) | Factor loadings: 0.06-0.13  variance explained: 63.5% (+) | α=.82-.90 (+) | NR |  | NR | CV: satisfaction and functional indices assess unique aspects of QoL: ≤0.43 sharing on average less than 18% of common variance as predicted. (+)  DV: significant differences reported between diagnostic groups across domains: (Wilk's λ=.93; F (16,2909) = 4.34***  (+) | Effect size between 0.4-0.82***  (102 days +/- 10.6) (+) |
|  | Lehman, 1988 |  |  | α= 0.44-0.88 (-) | NR | ICC= 0.29-0.98 (one week test-retest) (-) | NR | CV- correlations between two alternative forms of general life satisfaction scale: 0.63- 0.72****  (+)  DV- Between diagnosis:  Depression: *r* = -. 17 to -.56* Anxiety: *r* = -.25 to -.33**  (+) |  |
|  | Goodwin & Madell, 2002 |  |  |  | NR |  | NR | CV- strong correlations between Lehman’s QoL and QoL measures BIGALOW (Bigalow et al., 1991) and REHAB scale (five accomplishments) (Murray et al., 1998): 0.27-0.68** with QoL increasing with level of functioning.  Mood and QoL: 0.42-0.5*  (+)  DV- QoL scores significantly differed across residences: H = 11.64-20.6, df=2***  (+) |  |
| 3  Q-LES-Q | Bishop et al., 1999 | NR | Principle component analysis (PCA) revealed a four-factor model: 0.32- 0.45 Principal factor analysis (PFA) showed factors loadings: 0.35-0.50 (no variance explained)  (?) | α=.89-.96 (?) |  |  |  |  | NR |
|  | Ritsner et al., 2002 (10 items removed) | NR |  | α=.84-.95  (?) | Translated and back translated by a bilingual research assistant: Pilot tested N= 10. (?) | ICC=.47-.8 (two-week interval)  (-) | NR | CV:  Positive and Negative Symptom Scale (PANSS) (Kay et al., 1987):-0.24**  Montgomery-Asberg Depression Rating Scale (MADRS) (Fantino & Moore, 2009): -0.46**  No significant correlation with MARS-mania (0.13).  (+)  DV: Between groups  Patients: *r* =0.63***  Professionals *r* =0.39***  (+) | NR |
| 4  Q-LES-Q SF | Pitkanen et al., 2011 | NR | Classical test theory (CTT):  PCA: Factor loadings: 0.4-0.7 Variance explained: 56%  Kaiser Meyer Olkin (KMO): 0.889 Bartlett’s test of sphericity: χ 2 1117, df 105***  (+) | α=.89 (.40-74)  (+) | NR | NR | NR | CV: EQ-5D: ρ= 0.445*** (+)  DV: GAF (Global assessment of functioning): Differences in functioning: U=3357***  Diagnosis: U=4275 P=0.354 Not significant (NS)  (+) | NR |
| 5  LQOLP | Ritsner et al., 2002 (only 27 items considered) | NR |  | α=.42-92  (-) | Translated and back translated by a bilingual research assistant: Pilot test N= 10  (?) | ICC= .34-.82 (two week)  (-) | NR | CV:PANSS: -0.19*  MADRS: -0.34**  MRS mania: NS (0.05). As expected from literature. (+)  DV- Differences in QoL index for: Patients *r* =0.63*** Professionals *r* =0.39*** (+) | NR |
|  | Kaiser et al., 1997 | NR | Factor loadings: 0.51-.73  Variance explained: 15-42%  Eigenvalue: > 0.50  (-) | α=.73 (.69-.82)  (?) |  |  | NR | CV:  BPRS: β = -0.29 to -0.33 *r*2 = 0.09, F = 10.5***  BPRS total score: β = -0.25***  (+)  DV:  Settings: F= 0.83-6.10 NS- *p* <.001 across domains of QoL (Bonferroni corrections) (+)  Demographics and QoL: <10% shared variance across domains. (+) | NR |
|  | Gaite et al., 2000 | NR |  | α=0.87 (0.3-0.88)  (?) | Back translation completed: focus groups completed: NR: Pilot testing was completed: NR  (?) | ICC=0.82 (7-15 days) (+) | NR |  | NR |
|  | Hansson et al., 1998 | NR |  | α=0.93 (0.53-0.93)  (?) | Forward and back translations by first author: Pilot study N= 35 (?) | Pearsons *r* = 0.83-0.91*** (two weeks) (?) | NR |  | NR |
| 6  S-QOL-18 | Boyer et al., 2010 | NR | CTT  PCA: 8 factor structure  Factor loadings: >0.4  Variance explained: 78%  Inter-response theory (IRT):  Rasch model: INFIT 0.91-1.05  (+) | α=0.88 (0.72-0.84)  (+) | NR | ICC=0.73 (3-week interval)  (+) | NR | CV:  S-QOL-4: >0.88**  QLS: >0.5**  SF-36: 0.30–0.58**  QoLI: 0.28–0.58** (except finances NS)  with two of the three SQLS dimensions: -0.21 to -0.56*  EuroQol: 0.50**  PANNS: -0.28**  (+)  DV:  Age: 13 to -24**** (3 dimensions)  Gender: Male 58.7 and 65.7, female 49.4 and 58.4**(2 dimensions)  Education: (2 dimensions) 60.4-70.4**  Patient populations: 43.4- 64.6*  Duration of illness: (1 dimension) -0.21** in line with hypothesis.  (+) | Effect sizes >0.02* for multiple domains and index score over 6 months. (+) |
| 7  SQOL-41 | Auquier et al., 2003 | (+) | CTT  EFA eight factor structure.  Factor loadings: 0.46-0.84  Variance explained: 52% (+) | α= 0.94 (0.53-0.90)  (+) |  | ICC= 0.79 (0.64-0.79) (not reported, could be 30 days)  (+) | NR | CV:  Intercorrelation within scale: 0.24- 0.60***  Two domains not significant:  Psychological well-being and self-esteem: 0.66  Family relationships and resilience: 0.11 ( *p* = 0.14)  SQOL41: matching domains: psychological wellbeing, self- esteem, physical wellbeing  EuroQol: 0.48***  QoLI: 0.29-0.74***  SF-36: 0.29-0.61***  Unique areas: autonomy, resilience and sentimental life: not significantly correlated: as expected.  (+)  PANSS: -0.23**  Calgary Depression Scale for Schizophrenia (CDSS) (Addington et al., 1993): -0.39*** GAF: 0.27***  Clinical Global Impression of Severity (CGI) (Guy, 1976):-0.32*** As only one hypothesis deviated, overall score is (+).  DV:  Between groups: Outpatients score: M = 59.3 SD 17.8*  Inpatients score: 52.1 SD 18.3* (only *P* reported)  Length of illness: NS  Education: NS  Age: NS  (+) | effect size index score 0.44*  It was predicted that at least 0.2 indicated change over time.  (+) |
|  | Chou et al., 2010 |  |  | α = 0.93 (0.44-0.93) (+) | Forward translation and backward translation.  (?) |  | NR | CV:  SQLS-R4, WHOQoL-BREF, and RESE: 0.36–0.82*  (+)  DV: Mann–Whitney U test showed significant difference between subgroups based on number of hospitalisations and episodes U= 45.06** and 48.47*  (+) |  |
| 8  QLI | Angstman et al., 2009 |  | NR | NR | NR | NR | NR | NR | Effect size 0.34* (average 20 days). No predefined hypothesis. (+) |
| 9  QLIS | Franz et al., 2012 | (+) | NR | α= 0.63-0.88 with CTT described  (-) | NR | ICC=0.73-0.87 (7-10 days apart with stable non hospitalised patients) | NR |  | NR |
|  | Franz et al., 2012 |  | NR |  | NR |  | NR | CV:  WHOQOL-BREF: 0.30- 0.72 variance: 4.2-55.8%  Subjective Well-being Under Neuroleptic Treatment Scale (SWN) (Maurino, 2012): 0.14- 0.83 variance: 1.6-42.3%  Satisfaction scales: 0.18- 0.69 variance: 2.5-51.7%  (+) | NR |
| 10  How Are You? Scale | Katsavdakis et al., 1999  (study 2) | (+) | NR | NR | NR | NR | NR | DV: Between groups: Patients and normative sample: t=34.13***  (+) | Effect size 0.58*** (mean 16 days) (+) |
| 11  QLS | Nair et al., 1996 | (+) | NR | α = -1.5 - 0.8: negative value was reported for work/vocational rehab. No evidence of unidimensionality  (-) | NR | NR | NR | DV: Between groups: Staff: M = 2- 3.17 SD= 0.2- 1.27  Patient scores M= 1.86- 2.49 SD= 0.35- 1.07  *P*<0.05  (only *P* reported)  (+) | Responsiveness 6-month period between:  Old hospital: M= 0.98-2.43  New hospital: M= 0.94-2.26  No effect size reported. (-) |
| 12  Sf-36 | Newnham et al., 2007  (only subscales related to mental health) | NR |  |  | NR |  |  | DV: Between recovery groups on the Depression Anxiety and Stress scale (**DASS-21) (Lovibond, 1995): Depression**: F(3,1533) = 95.51** **Anxiety**: F(3,1534) = 49.87** **Stress**: F(3,1525) = 67.62** Recovered patients (via SF-36) also had **higher Q-LES-Q scores**: F(3,858) = 57.30**  **lower clinician-rated HONOS scores:** F(3,1524) = 10.60**  No hypothesis stated. (+) | (2 year period) **RCI: 1.96**.** Effect sizes were not calculated (+) |
|  | Pukrop et al., 2003 | NR |  | α= .79 -0.83  (+) | NR |  |  | CV:  MSQoL: 0.61- 0.84**  PANSS: NS  Hamilton Depression Rating Scale (HAM-D) (Hamilton, 1960): -0.17 to -0.53*  No hypothesis made.  (+)  DV: Between three groups of patients:  Admission: F= 38.4-230.2*** Discharge: F= 12.4-132.9***  (+) | Responsiveness admission and discharge (minimum time of 3 weeks)  Depressed patients: t= 3.51-9.39*** (2 NS results)  Schizophrenic patients: t= 2.17-4.19*** (4 domains) No effect sizes.  (+) |
|  | Nishiyama et al., 2009 | NR |  | MMSE<24 α= 0.3-0.91  MMSE <25 α= 0.26-0.88  MMSE <28 α= 0.63-0.9.  (-) | NR |  |  | CV: Convergent validity was assessed by the percentage of items correlating ≥ 0.40 with their hypothesized domain, using multi-trait scaling analysis. Correlation rates ranged from **20% to 100%** across domains and MMSE groups. Convergent validity declines with increasing cognitive impairment. (+)  DV: Percentage of items correlating more strongly with their own scale than with others. **MMSE < 24:** 14%–100% **MMSE < 25:** 29%–100%. **MMSE < 28:** 75%–100%**.** (+) |  |
|  | Su et al., 2014 | NR | CFA  CFI: 0.918 Incremental fit index (IFI): 0.919  TLI: 0.885  RMSEA: 0.145  SRMR: 0.073 COSMIN guidelines will accept CFI and SRMR scores.  (+) |  | NR | ICC= .87 (0.48-0.81) (2 weeks) (+) | Measurement error SEM= 10.03% smallest real difference (SRD) = 27.8% (acceptable)  (+) | CV:  WHOQOL-BREF: traits and methods correlated (CFI = 0.982, IFI=0.982 TLI 0.970 RMSEA = 0.068, SRMR = 0.050)  (+)  DV: Between groups:  Taiwan general population: M= 524.4 +/- 144.10  Patients: M= 637.51  (*p* <0.01)  only *P* reported)  (+) |  |
|  | Tunis et al., 1999 | NR | CTT  EFA: Factor loadings: 0.46-0.70 Variance explained: 56%  (+) | α= 0.9 (0.76-0.91)  (+) | NR |  |  | CV: mental health component and:  BPRS: -.31***  CGI: -.15***  MADRs: -.55***  Physical component: NS  (+)  DV:  Age: NS mental health component  *r*=-0.19*** physical health  Between general population: M= 60.9 - 84.2  Schizophrenic patients means:  M= 41-82.1***, except bodily pain. (+) | Responsiveness (6 weeks)  Mean change= 1.31- 14.23 NS to *p* <0.0001. No effect size calculated.  (+) |
| 13  WHOQOL-BREF | Su et al., 2014 | NR | CFI: 0.967  IFI: 0.967  TLI: 0.900  RMSEA: 0.216 SRMR: 0.037  (+) |  | NR | ICC= 0.81-0.88 (2 weeks) (+) | Measurement error: SEM total: 5.55% SRD total score: 15.4%  (+) | CV:  SF-36: traits and methods correlated (CFI = 0.982, RMSEA = 0.068, SRMR = 0.050)  (+)  DV- Taiwan general population WHOQOL-BREF: M= 52.82 8.29 Patient population.: M= 57.35  *(p* <0.01)  (only *P* reported)  (+) | NR |
|  | Oliveira et al., 2016 | NR | CTT  PCA: Factor loadings: 0.55-0.9  Variance explained: 59.51%  CFA: X2=180.262***  CFI: 0.955  GFI: 0.914  TLI: 0.942  RMSEA: 0.050 SRMR: 0.055  (+) | α= 0.68-0.85  (-) | NR |  |  | CV: All domains predicted QOL significantly: General QoL adjusted: 0.51*  (+) | NR |
|  | Norholm & Bech, 2006 | NR |  | Loevinger’s homogeneity coefficients (0.34–0.47 in patients) Cronbach’s alpha was not calculated.  (?) | NR |  |  | DV:  Schizophrenic population total QoL: M= 25.6  General population total QoL: M= 6.8  Schizophrenic population gender differences: *p* <0.05  Gender differences between QoL and MDI scores: male: M= 22 Female: M= 30 (*p* <0.01)  (only *P* reported)  (+) | NR |
|  | Chan et al., 2003 | NR |  |  | NR |  |  | CV:  BPRS: -0.1 to -0.23* (finance not significant) (+)  GAS: Psychological domain: 0.14*  Environment domain: 0.15*  Total negative life events: -0.42 to -0.23*  Subjective well-being sociodemographic and clinical variable: F= 3.914- 8.725****  (+)  DV: Between populations: Life satisfaction: F=7.74*** Environment: F=3.53*  all other domains were NS.  (-).  Education: Life satisfaction: -0.25****  Environment: -0.15*  All other domains don’t correlate  (-) | NR |
|  | Van de Willige et al., 2005 | NR |  |  | NR |  |  | CV: EQ-5D: 0.55-0.58***  PANSS: -0.54***  Auditory Hallucination Rating Scale (AHRS) (Haddock et al., 1999) negative contents: -0.31** distress: -0.54***  Social functioning measured by Groningen Social Disabilities Schedule (GSDS) Wiersma et al., 1990): -0.57***  (+) | Significant changes in treatment group: t = 2.11*. No effect size  (18 months)  (+) |
| 14  MSQOL | Pukrop et al., 2003 | NR | NR | α= 0.83- 0.87, no evidence of unidimensionality provided. (?) | NR | NR | NR | CV: Sf-36: 0.61- 0.84**  There were no significant correlations with negative and positive symptoms (PANSS positive and negative symptoms).  HAM-D: -0.18 to -0.51* (role physical NS)  (+)  DV: Comparison between patient groups: Admission: F= 40.8-364.5***  Discharge: F= 5-46.8*** (+) | Admission and discharge (minimum time of 3 weeks)  Depressed patients: t = 3.79-16.60***  Schizophrenic patients: t = 0.98- 7.63*** (3 domains NS) no effect size.  (+) |
| 15  EQ-5D | Pikanen et al., 2011 | (+) | PCA  KMO: 0.701  Bartletts test of sphericity: χ 2 166, df 10,***  EFA: factors loadings: 0.52-0.708 Variance explained: 41.3%  (-) | α= 0.63 (0.530-0.63)  (-) | NR | NR | NR | CV- Q-LES-Q: ρ =0.445*** (+)  DV: GAF: Lower functioning: Median score 0.64***  Higher functioning: Median score 0.89***  Diagnosis: NS  (+) |  |
|  | Van de Willige et al., 2005 |  |  |  | NR | NR | NR | CV- WHOQOL: 0.55-0.58***  PANSS factors: -0.31 to -0.47****  AHRS negative contents and visual analogue scale (VAS) score: -0.46***  Negative contents and time trade off (TTO): NS  Distress and TTO and VAS score: -0.25 to -0.50***  Social functioning measured by GSDS: -0.29 to -0.41*  (+) | (18 months): t = 2.37* No effect size.  (+) |
| 16  MHQOL | Ebadi & Rezaiye, 2025 | (+) | CTT  EFA and CFA  59.45% variance explained with one factor removed. GFI (0.90), CFI (0.97), IFI (0.97), NFI (0.95), PNFI (0.64), RMSEA (0.14), CMIN/DF (3.5), and RMR (0.049). Factor loadings (0.39-0.948) scree plot used (+) | α= 0.876  (+) | Translation (WHO): N= 10 Loop analysis: N= 30  (*r* <0.3).  (?) | ICC= 0.97 (two-week interval)  (+) | SEM= 0.14  (+) | NR | NR |
| 17  SF-12 | Soysal Gunduz et al., 2021 | NR | CTT EFA/PCA  KMO: 0.83  Bartletts: X2 =938.30****  Factor 1 eigenvalue of 5.58 representing 46.57% of variance.  Factor 2 eigenvalue of 1.57 representing 13.14% of variance. 59.7% cumulative variance  (+) | Physical health component summary α= 0.80 (0.32-0.73) mental health component summary α= 0.88 (0.60-0.78)  (+) | NR | NR | NR | CV: NHP health profile: -0.03 to -0.58 (no *p* reported)  (?)  DV: Surgical group: M= 46- 80.5  Psychiatric group: M= 41.7- 65, rheumatology group: M= 47.9-88.9  (*p*<0.0001)  (only *p* reported)  (+) | NR |
| 18  ReQoL | Keetharuth et al., 2018 | (?) OutPatients | CFA  RMSEA: ≤0.08  CFI: >0.95  IRT: *r* = 0.98 Expected A Posteriori (EAP): 0.95.  CFA: 6 factor model  EFA: 2 factor solution: 0.8.  IRT analysis: *r* =0.98  MISFIT: *p* <0.05  DIF (age gender, ethnicity, gender)  (+) | REQOL 10: α =0.92  REQOL20: α=0.96  (+) | Translatability assessment was conducted and DIF calculated  (?) | ICC: 2-week interval  ReQoL 10: ICC= 0.85 ReQoL 20: ICC= 0.87 | NR | CV: Short Warwick-Edinburgh Mental Health Wellbeing Scale (SWEMWBS) (Stewart-Brown et al., 2009) and Clinical Outcomes in Routine Evaluation (CORE-10) (Barkham et al., 2013): >0.8**  REQOL 10 and REQOL 20: 0.98**  (+)  DV: Between general population and patient population (hypothesis made).  REQOL 10: SES= 0.93  REQOL 20: SES= 1.05  (+) | Effect size: small <0.2 to moderate <0.5 change over time. (no *p* reported) (+) |
| 19  SQLS | Kaneda et al., 2002 | NR | CTT  PCA: 55.8% variance accounted for and factor loadings greater than 0.5 (three domains), some factors failed to load  (?) | α = 0.73-0.93  (?) | Forward and back translation: Pilot tested: N= 5  (?) |  |  | CV-  SF36: -0.680*** and -0.375**  No significant correlation with WHOQOL and Drug Induced Extrapyramidal Symptoms Scale (DIEPSS) (Peljto et al., 2017)  Three out of four hypothesis confirmed (75%)  (+) |  |
|  |  |  |  |  |  |  |  |  |  |
|  | Kuo et al. 2007 | NR |  | α= 0.84-0.92 for both domains (two domains)  (+) |  | ICC= 0.844 for both domains (two weeks) | SEM= 86% met criteria for changes smaller than 2 SEM (+) | CV: SF-36: -0.25 to -0.65*  Negative correlations due to different polarities of the scores on the two instruments. expected correlations.  Psychosocial domain: -0.67  (+) | Two-week period: Responsiveness coefficient: (RC) 0.55 and 0.03 improved and 0.27 and -0.09 in deteriorated  (?) |
|  | Kuo et al., 2009 | NR | EFA (three domains)  KMO: 0.87  Bartlett: 1763.12***  PCA varimax rotation: 68.1% variance explained factor loadings all above 0.3  (+) |  |  |  |  | CV: PANSS and GAF: NS  T-LQOLP: psychosocial domain: -0.46** and -0.22*  Vitality domain*:* -0.54** and -0.18*  (+) |  |
|  | Martin & Allen, 2007 | NR | CFA:  Two factor correlated  CFI (0.95)  TLI (0.98)  RMSEA (0.11)  SRMR (0.94)  (-) | α = 0.83-0.96,  (+) |  |  |  | CV:  HADS A: 0.89**  HADS D: 0.70**  (+) |  |
|  | Su et al., 2017 | NR | CFA (three domains)  CFI (0.967),  TLI (0.964),  RMSEA (0.068),  SRMR (0.072),  3-dimensional construct all factor loadings significant factor loadings for 33 item model 0.47-0.76% variance explained  (+) |  |  |  |  | CV:  WHOQOL BREF*:*-0.26 and -0.69**  (+) |  |
| 20  QOL-BD | Michalak et al., 2010 | (+) | Latent structure factor analysis.  Factor loadings >0.3 KMO: 0.92  Bartlett’s test of sphericity: v2 = 9075.85*** 12-factor extraction explained 71% of the shared variance.  (+) | α= 0.79-0.93  (+) | NR | Internal reliability test-retest 0.46-0.77 (7 to 10 days)  (-) | NR | CV-  Expected correlations were found between QOL measures (SF36, QLESQ, SWL) and symptom severity measures (PANSS HAMD)  HAM-D: -0.62 (b=)0.323, t=)2.04*  Young Mania Rating Scale (YMRS) Young et al., 1978): 0.18  SF-36: 0.63  Q-LES-Q: 0.89 (no *p* reported)  In line with hypothesis.  (+) | NR |
| 21  Q-LES-Q-18 | Ritsner et al., 2005 | NR | Varimax rotation factor analysis  Four factors were identified on the highest eigenvalues (>1.0):  These factors accounted for 31.4%, 26.3%, 22.7% and 19.5% of the total variance.  (+) | α= 0.96 (0.74-0.97) inpatient sample  (+) | NR | ICC=0.91 (2 week) (outpatients)  (not rated for inpatient) | NR | CV-  LQOLP: 0.33- 0.51***  QLIS: 0.51-0.64***  Q-LES-Q: 0.55 to 0.64***  (+)  DV: Between patients and healthy controls: F=42.0***  (+) | NR |
| 22  Henrich’s Quality of Life Scale | Simon-Abbadi et al., 1999 | NR | CTT Factor analysis varimax rotation  53.5% variance explained. Negative factor loadings reported.  (-) | α = (0.9) 0.6-0.9  (-) | NR | inter rater reliability, no time interval reported ICC- 0.72-0.79  (+) | Weighted Kappa= 0.1-0.7  (?) | CV: With subjective quality of life assessment: 0.3**  GAF: 0.56***  (+) | NR |

**Table 3**

*Content Validity*

| OMI | Reference | Clear construct  (definition) | Origin of construct  (theory or evidence base) | Target population for which the PROM was developed | Intended  context of use | Content Validity  Relevance, comprehensibility, comprehensiveness | | | |
| --- | --- | --- | --- | --- | --- | --- | --- | --- | --- |
|  |  |  |  |  |  | Pilot testing | Patients | Professionals | |
| 1 | Anderson et al., 2002 | Objective QoL: based on functional access to resources | Response to requests for a briefer LQOLI. | NR | NR | NR | NR | NR | |
| 2 | Russo et al., 1997 | Personal characteristics, objective and subjective QoL. Multidimensional definition of QoL | National QoL studies and existing QoL measures | Mentally unwell patients | Inpatient and outpatient mental health |  | NR |  |  |
|  | Lehman, 1988 |  |  |  |  | Comprehensibility: Interview format (45 minutes), pilot trials with target population.  No description of method or design. | NR | Comprehensiveness: selection of items based upon national quality of life studies. Items were pulled from existing instruments. Comprehensibility: modified to increase | |
| 3 | Goodwin & Madell, 2002 | QoL  measures of the degree of enjoyment and satisfaction experienced by subjects in areas of daily functioning. | Built on previous research of HRQoL, little research in QoL within psychiatry. | Medical and psychiatric disorders. Developed for use with depressed patients | NR | NR | NR | NR | |
|  | Bishop et al., 1999 |  |  |  |  |  |  |  |  |
| 4 | Ritsner et al., 2002 (10 items removed) | QoL enjoyment and satisfaction experienced by patients with mental disorders | NR | Patients with mental disorders | NR | NR | NR | NR | |
| 5 | Pitkanen et al., 2011 | Defined by objective and subjective life circumstances. HRQoL, multidimensional. | Developed from LQOLI in the context of deinstitutionalisation. | Schizophrenic patients- mental illness related | Multiple psychiatric subpopulations. Mixed settings. | NR | NR | NR | |
|  | Ritsner et al., 2002 (only 27 items considered) |  |  |  |  |  |  |  |  |
|  | Kaiser et al., 1997 |  |  |  |  |  |  |  |  |
|  | Gaite et al., 2000 |  |  |  |  |  |  |  |  |
|  | Hansson et al., 1998 |  |  |  |  |  |  |  |  |
| 6 | Boyer et al., 2010 | QoL in schizophrenia- multidimensional | Condensed version of S-QOL-41 | Patients with schizophrenia | NR | NR | NR | NR | |
| 7 | Auquier et al., 2003 | Subjective QoL, multidimensional. | Somatic medicine and human sciences. | Patients with schizophrenia | Inpatient and outpatient mental health | Comprehensibility: Item generation, 90 items: pilot tested with target population N= 40  Relevance: Producing 83 question format. A second survey further reduced the items excluded irrelevant items. | Relevance: Videotaped interviews with Schizophrenic in and outpatients: N=20 Trained interviewer and interview guide used.  Relevance: Cognitive debriefing N=27, asked to point to important domains | Comprehensiveness: Involvement of a steering committee who confirmed the content was meaningful. | |
|  | Chou et al., 2010 |  |  |  |  |  |  |  | |
| 8 | Angstman et al., 2009 | Subjective QoL: important needs, goals, and wishes, and fulfilment. | Developed from LQOLI | Social phobia, generalised anxiety disorder, and depression | A range of settings | NR | NR | NR | |
| 9 | Franz et al., 2012 | Subjective QoL emphasising context. | Identifies that QoL needs a better theoretical foundation. | Schizophrenia | Hospital: day, in and outpatient | Comprehensibility: Pilot version developed and tested. Consideration to comprehensiveness and difficulty of questions. Final version tested. | Relevance: Open-ended interviews with schizophrenic patients from different care settings to identify relevant items | Comprehensiveness: Experts in the area paraphrased each item and excluded redundant items. | |
|  | Franz et al., 2012 |  |  |  |  |  |  |  | |
| 10 | Katsavdakis et al., 1999  (study 2) | Considering experiences that the service user themselves finds important and the negative impact of mental health (subjective QoL) | Developed to address both the concerns of the consumer movement and the necessity of providing objective outcomes - reference to biopsychosocial model in differing context | Psychiatric patients | Inpatient and outpatient | Relevance: Pilot tests reduced initial 90 items that had been generated from the FACE domains to 62. The final version was then pilot tested. | Comprehensiveness and relevance: Focus groups inpatient and outpatient. To feedback on comprehensibility and removing irrelevant items and altering content reduced to 55 items. | Comprehensibility: Understanding from research to develop a scale that is more comprehensible. | |
| 11 | Nair et al., 1996 | QoL in chronically mentally ill (subjective) | Based on the material and social world together with subjective experiences as contributing to the individual patient’s perception of their QoL | Chronically mentally ill. | Rehabilitation long stay psychiatric inpatients | Relevance: Pilot test final form: N=50 patients, reduced to exclude religion. | NR | Relevance and comprehensibility: Medical and nursing staff had a group discussion and provided recommendations. Following these 8 life domains were selected. | |
| 12 | Newnham et al., 2007  (only subscales related to mental health) | QoL- objective and subjective  as defined by the world health organisation (WHO) | Originated from health insurance and development in the article to apply to psychiatric population- The health insurance experiment (Brook et al., 1983). | Generic instrument for medical outcome measurement  General adult population. | Various healthcare setting, in and outpatient. | NR | NR | NR | |
|  | Pukrop et al., 2003 |  |  |  |  |  |  |  |  |
|  | Nishiyama et al., 2009 |  |  |  |  |  |  |  |  |
|  | Su et al., 2014 |  |  |  |  |  |  |  |  |
|  | Tunis et al., 1999 |  |  |  |  |  |  |  |  |
| 13 | Su et al., 2014 | QoL as defined by WHO. Objective, subjective and global level of functioning. | QoL is the result of a patient’s continuous efforts to adapt to both internal (illness related) and external circumstances. | Adults with disease or impairment and healthy adults | Across different contexts | NR | NR | NR | |
|  | Oliveira et al., 2016 |  |  |  |  |  |  |  |  |
|  | Norholm & Bech, 2006 |  |  |  |  |  |  |  |  |
|  | Chan et al., 2003 |  |  |  |  |  |  |  |  |
|  | Van de Willige et al., 2005 |  |  |  |  |  |  |  |  |
| 14 | Pukrop et al., 2003 | QOL as a multidimensional measure | Created from the combination of items from several questionnaires (LQLP, NHP, Psychological general well-being schedule, questionnaire for everyday living, questionnaire for the state of health, satisfaction questionnaire, SF-36, sickness impact profile). | Healthy controls, patients with depression and patients with schizophrenia | Designed for a psychiatric setting | NR | NR | NR | |
| 15 | Pikanen et al., 2011 | A preference-based measure of HRQoL (subjective QoL) | Developed by the EuroQoL group. | Measure of QoL in various diseases.  A generic QoL measure, that has been used in a range of population surveys, including the English national health survey. | To be used in the evaluation of diﬀerent healthcare interventions | NR | NR | NR | |
|  | Van de Willige et al., 2005 |  |  |  |  |  |  |  |  |
| 16 | Ebadi & Rezaiye, 2025 | Based on WHO definition of QoL. | Origin of scale has stemmed from impact of QoL after psychiatric disorder. | Mental health | NR | Pilot test, Loop analysis: N= 30 patients | Relevance and comprehensibility: Feedback: N=10 psychiatric patients | Comprehensibility: Feedback on wording and scoring: : N=10 professionals  Relevance: N=10 professionals provided ratings on relevance. | |
| 17 | Soysal Gunduz et al., 2021 | HRQoL- health status of a patient was considered beyond their illness and objective criteria. | HRQoL scales were initially developed for research purposes. Derived from SF36 | Physical, mental health and general population. | Across multiple contexts | NR | NR | NR | |
| 18 | Keetharuth et al., 2018 | Based on aspects of QoL that matter to mental health service  Users (subjective QoL) | Based on the following framework: Connectedness, Hope, Identity,  Meaning and Empowerment (CHIME) and O’Connell (2014) framework. | Mental health | Multiple contexts including secondary care (in and outpatient) primary care, increasing access to psychological therapies (IAPTs), general population, CAMHS. | Relevance: N=19 outpatients’ interview to generate 7 themes. | Comprehensiveness, comprehensibility and relevance: Qualitative interviews with adult from CMHT and young people added 12 new items and eliminated 39 items and rephrasing. | Comprehensiveness:  Systematic review of literature: 1597 items were generated and reduced to 88 items.  Scientific group considered all items and REQOL was developed. Feedback sought: N= 33 clinicians. | |
| 19 | Kaneda et al., 2002 | QoL- developed in response to lengthier scales and to be self-report  Subjective QoL, based on WHO definition. | Brief self-report QoL measure specific to  people with schizophrenia, Wilkinson et al. (2000)  developed a new scale, the SQLS.  Derived from Wilkinson et al. (2000) SQLS | Schizophrenic patients | Clinical trials | NR | NR | NR | |
|  | Kuo et al. 2007 |  |  |  |  |  |  |  |  |
|  | Kuo et al., 2009 |  |  |  |  |  |  |  |  |
|  | Martin & Allen, 2007 |  |  |  |  |  |  |  |  |
|  | Su et al., 2017 |  |  |  |  |  |  |  |  |
| 20 | Michalak et al., 2010 | QoL as defined by WHO  (Subjective experience) | Developed from the need for additional forms of assessment outside of objective measures to measure treatment outcome of recovery. Developed as disorder specific scales can potentially be more sensitive to change. | Bipolar | Clinical trials of pharmacological or psychosocial treatment interventions,  longitudinal monitoring, or routine clinical care | Comprehensibility: Pilot questionnaire: N= 225 participants.  Refinements made before final study. | Relevance and comprehensiveness: Interviews: N=35 in and outpatient. Tape recorded and to saturation. | Comprehensiveness: N= 5 caregivers N= 12 researchers participated in interviews. Data triangulation with literature. | |
| 21 | Ritsner et al., 2005 | HRQoL | Derived from QLESQ, to reduce the items to a core subset. | BD, depression, PMDD, and acute coronary syndrome, psychoses, PTSD, postsurgical states,  schizophrenia | Used in QoL outcome studies of  mentally disabled patients | NR | NR | NR | |
| 22 | Simon-Abbadi et al., 1999 | HRQoL- no consensus definition reported in paper. | Biopsychosocial perspective  For use with schizophrenic deficit syndrome, and as a measure of  psychosocial functioning | Schizophrenia specific | Psychopharmacologic treatments, predominantly in  outpatients | NR | NR | NR | |
